# Plasmon-mediated dehydrogenation of the aromatic methyl group and benzyl radical formation†

**DOI:** 10.1039/d3sc05847f

**Published:** 2023-11-28

**Authors:** Jianghao Zhou, Jing Guo, Govinda Ghimire, Alexander M. Mebel, Shuai Chang, Jin He

**Affiliations:** a The State Key Laboratory of Refractories and Metallurgy, School of Materials and Metallurgy, School of Chemistry and Chemical Engineering, Wuhan University of Science and Technology Wuhan 430081 China schang23@wust.edu.cn; b Department of Physics, Florida International University Miami Florida 33199 USA jinhe@fiu.edu; c Department of Chemistry and Biochemistry, Florida International University Miami Florida 33199 USA; d Biomolecular Science Institute, Florida International University Miami Florida 33199 USA

## Abstract

Plasmonic molecular junctions can harvest visible light and effectively catalyze chemical reactions. The strong light field concentrated in the plasmonic junction also enables the application of surface enhanced Raman spectroscopy (SERS) to probe the catalyzed chemical reactions *in situ* and in real time down to single-molecule resolution. The benzyl radical produced from the aromatic methyl group through the dehydrogenation reaction is an important precursor for a large variety of reactions. Here, we used time-resolved SERS to conduct a mechanistic study of the plasmon-driven dehydrogenation reaction of the aromatic methyl group under ambient conditions under the illumination of red light on the apex of a gold nanoelectrode. Transient spectral changes with intensity bursts are frequently observed. Based on density functional theory and picocavity based local electric field enhancement calculations, they result from the plasmon mediated dehydrogenation reaction of aromatic methyl groups. The dehydrogenation reaction produces a benzyl radical, which is consequently converted to a benzyl anion. The benzyl anion is stabilized through strong interactions with gold, leading to the formation of dynamic gold adatoms and picocavities. In addition to the benzyl anion, we found spectral evidence that the benzyl radical generates dimers through a self-reaction. Furthermore, we demonstrated that the dehydrogenation reaction could be facially modulated by changing the electrode potential, which is attributed to the modulated inductive effect.

## Abstract 2

A plasmon-driven deprotonation reaction of the aromatic methyl group can occur in aqueous solution under the illumination of red light. The reaction produces a benzyl radical and anion, and dimers through a self-reaction.

## Graphical abstract

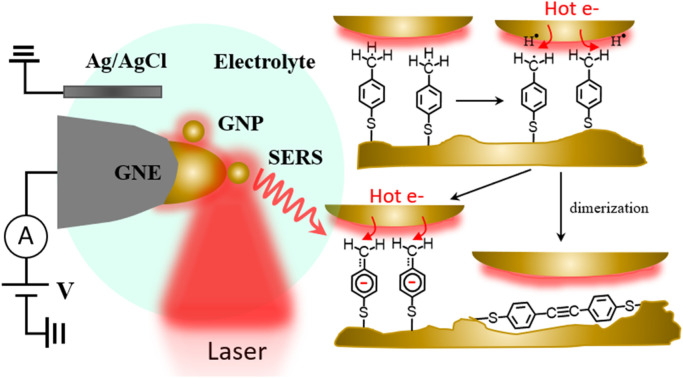

## Introduction

As demonstrated in recent years, the plasmonic nanostructures of noble metals have become effective nanoscale chemical reactors for green synthesis by harvesting solar light energy to trigger plasmon-mediated chemical reactions (PMCRs) under mild conditions.^1–5^ The nonradiative decay of surface plasmons excited on the metal surface induces local heating and energetic charge carriers (hot electrons and holes).^6–10^ They can catalyze and lower the energy barrier of chemical reactions of adsorbed molecules. The greatly concentrated light field inside the plasmonic nanostructures also enables strong signals in surface enhanced Raman spectroscopy (SERS),^11,12^ which has been used to monitor many PMCRs in operando down to the single-molecular level, providing mechanistic insight into the photochemical process.^1^

Radicals play an important role in organic synthesis.^13^ As a relatively stable aromatic radical, the benzyl radical (C_7_H_7_) is an important precursor for many reactions in combustion, atmospheric chemistry, astrochemistry, and biochemistry.^14–18^ The resonantly stabilized benzyl radical can be formed through the dehydrogenation reaction of aromatic methyl groups (*i.e.*, toluene).^19^ Owing to the inductive effect,^20^ the aromatic methyl group with a hyper-conjugated structure becomes more reactive.^21,22^ The benzyl radical has been formed from toluene by UV light irradiation,^23^ combustion at high temperature^24^ and means of transition metal catalysts and strong oxidants.^15^ Continued efforts have been made to develop new approaches to form benzyl radicals under mild conditions, such as electrochemical reduction of benzyl chloride^25,26^ and mediated electrolysis.^27,28^ The PMCR may provide a new green approach to produce benzyl radicals from aromatic methyl with visible light under extremely mild conditions.

**Fig. 1:**
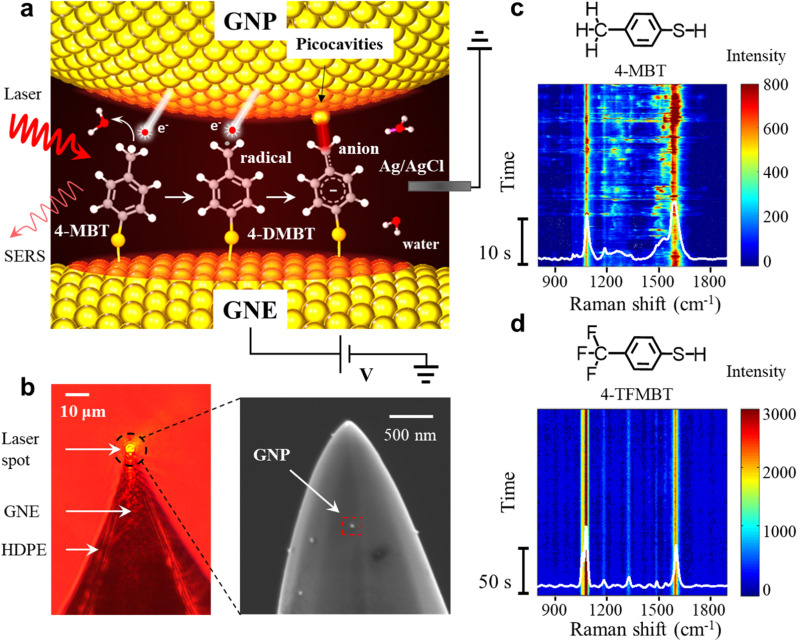
(a) SERS experimental setup and the schematic representation of the plasmon mediated reaction of the 4-MBT molecules in the GNE-GNP junctions, sequentially resulting in a benzyl radical and anion. (b) Left: optical microscope image of a HDPE insulated GNE with the laser beam focused on the apex. Right: SEM image of a 4-MBT modified GNE apex (without HDPE coating) with attached GNPs. (c and d) Representative SERS time trajectory of 4-MBT (c) and 4-TFMBT (d).

Herein, we studied a plasmon-mediated reaction involving the dehydrogenation of the methyl group of 4-methylbenzenethiol (4-MBT) within self-assembled plasmonic molecular junctions located at the apex of a gold nanoelectrode (GNE) in an aqueous solution. We monitored the reaction *in situ* by SERS at the single-molecule level (see Fig. 1a). Frequently fluctuating spectral changes were observed in the time-resolved SERS spectra under the illumination of red light (632.8 nm laser). Aided by density functional theory (DFT) calculations, a significant fraction of those fluctuating signals was found to be linked to the ‘picocavity’^29,30^ enhanced signatures of deprotonated 4-MBT (4-DMBT) in the benzyl anion form. Based on DFT calculation, these benzyl anions are likely converted from benzyl radicals, which are produced from a Plasmon-mediated dehydrogenation reaction of the 4-MBT methyl group. The ‘picocavities’ originating from gold adatoms^31^ caused extremely high local electric field enhancement (LEFE) on the atomic scale due to the lightning-rod effect, thereby producing frequently fluctuating transient signals at the single-molecule level. We attributed the high occurrence rate of the ‘picocavity’ to the stronger interaction between the benzyl anion and gold adatoms. In addition, the benzyl radicals can induce a dimerization reaction, the product of which is observed. By manipulating the inductive effect with an externally oriented electric field,^32,33^ we have also successfully modulated the reaction by facilely changing the applied potential on the GNE.

## Experimental methods

### Chemicals

4-MBT was purchased from Aladdin. 4-(Trifluoromethyl)benzenethiol(4-TFMBT) and benzenethiol (BT) (≥99%) were purchased from Sigma-Aldrich. Phosphate buffered saline (PBS) powder (pH 7.3–7.5) and absolute ethanol (200 proof) were purchased from Fisher Scientific. Potassium ferrocyanide (98.5%, analysis grade) was purchased from Acros Organics. Citrate stabilized 40 nm diameter gold nanoparticles (GNPs) were purchased from Ted Pella, Inc. All the aqueous solutions were prepared using deionized water (∼18 MΩ) purified with a Purelab system of ELGA/Siemens.

### SERS measurements on the GNE apex

The details of the SERS measurement setup have been reported previously.^34–36^ Briefly, SERS is performed on a home-built Raman microscopic setup based on a Nikon Ti–U microscope. A 632.8 nm HeNe laser was focused on the GNE tip (see Section S2 of the ESI† for GNE fabrication) placed in a liquid cell. The beam radius is about 3 μm, which gives an approximate power density of 8.14 μW μm^−2^ while focused on a typical GNE apex. The time-resolved SERS trajectories were collected with a time resolution of 50 ms by using a CCD camera (PIXIS 100B_eXcelon, Princeton Instrument). The spectral resolution is about 2 cm^−1^. An Axon 200B patch-clamp amplifier (Molecular Devices Inc., CA) in voltage-clamp mode was used to supply the electrode potential and amplify the current. A 10 kHz Bessel low-pass filter was typically used for applying electrode potentials to the GNE. The obtained data were analyzed by using custom Labview (National Instruments) and Matlab (MathWorks Inc.) programs.

The GNE is used as the working electrode and the Ag/AgCl wire electrode is used as the quasi-reference electrode. All the measurements are performed at room temperature in the electrolyte. The typical electrolyte is 10 mM PBS (pH 7.4). Initially, the liquid cell contains GNPs with a concentration of 150 pM. After stable peaks are observed in the SERS spectra (typically 5 min), the solution in the liquid cell is replaced with a new 10 mM PBS solution without GNPs. Normally, the GNP number density on the GNE apex is about 4–5/μm^−2^ based on the SEM images.

### Data analysis

The SERS experiments were usually repeated 2 to 3 times. The data collection time for each GNE was about 100 minutes. The SERS data were processed by using custom programs written in MATLAB and LabVIEW, and by using OriginPro software. The SERS spectral baselines fitted by using the asymmetric least square method were subtracted from the original SERS spectra. The representative fitted baselines are shown in Fig. S3.† To select the dynamic signals, we only picked the signals with their intensity at least 5 times higher than the baseline noise (typical standard division is 50 counts) and having clear starting and ending points.

### DFT calculations

DFT calculations, including the energy calculation, the Raman spectra and the atomic structural optimization of the molecule–gold cluster complexes, were carried out with Gaussian09 (see Section S6 of the ESI†). The wb97xd exchange-correlation functional and 6-311G(d,p) basis set were used for molecules, and the MWB60 basis set and effective core potential were used for gold atoms. For the LEFE calculation, the details of the method have been reported in previous work.^37^

## Results and discussion

### The dehydrogenation of the aromatic methyl group and the formation of picocavities

In the SERS measurements, the laser beam is focused on the exposed GNE apex out of the high-density polyethylene (HDPE) insulation (Fig. 1b). A 4-MBT plasmonic molecular junction is formed on the 4-MBT modified GNE apex due to the adsorption of GNPs from the bath solution (named NPoNE structures).^34,37^ The SERS intensity is very weak before GNP adsorption and increases gradually with the increased number of adsorbed GNPs. After the SERS intensity reaches its plateau, suggesting that the number of adsorbed GNPs are saturated on the GNE apex, we replenish the liquid cell with a new 10 mM PBS solution without GNPs for measurement. Fig. 1b shows the scanning electron microscope (SEM) image of a 4-MBT modified GNE apex with adsorbed GNPs. From our estimation, the SERS signal is typically contributed by slightly over 250 molecules in the hot spots of about 30 NPoNE structures on one GNE apex (see S2 of the ESI†).

Fig. 1c shows a typical SERS time trajectory in intensity heatmap format and the time-averaged SERS spectra from a GNE apex. The two major peaks near 1080 cm^−1^ and 1590 cm^−1^ are from the stretching modes of C_ring_–S (coupled with in plane benzene ring breathing mode) and C_ring_–C_ring_, denoted by *v*(C_ring_–S) and *v*(C_ring_–C_ring_), respectively.^34,35^ Interestingly, we often observe the frequent intensity bursts in the heatmap at a typical excitation laser power density of 8.1 μW μm^−2^. Each burst event usually lasts less than 1 s. Due to the high synchronization for these transiently fluctuating peaks, these signals are likely from individual molecules. In the average spectrum, a broad peak centered at 1530 cm^−1^ is obvious, which reveals that some of the events also contain a new peak around 1530 cm^−1^. To explore the origin of the spectral fluctuations, we performed control experiments with 4-TFMBT and BT molecules. The representative SERS time trajectory and its average spectrum for 4-TFMBT are shown in Fig. 1d. We did not observe the spectral fluctuations for 4-MBT under the same experimental conditions. Very stable spectra are also observed for the BT molecule (S4 of the ESI†). Therefore, the fluctuations were attributed to the methyl group of 4-MBT. The Raman spectrum of 4-MBT powder is also stable without a fluctuating peak near 1530 cm^−1^ (see S1 of the ESI†). Therefore, the peak near 1530 cm^−1^ is attributed to the plasmon-activated methyl group inside the hotspots of the NPoNE structures.

**Fig. 2:**
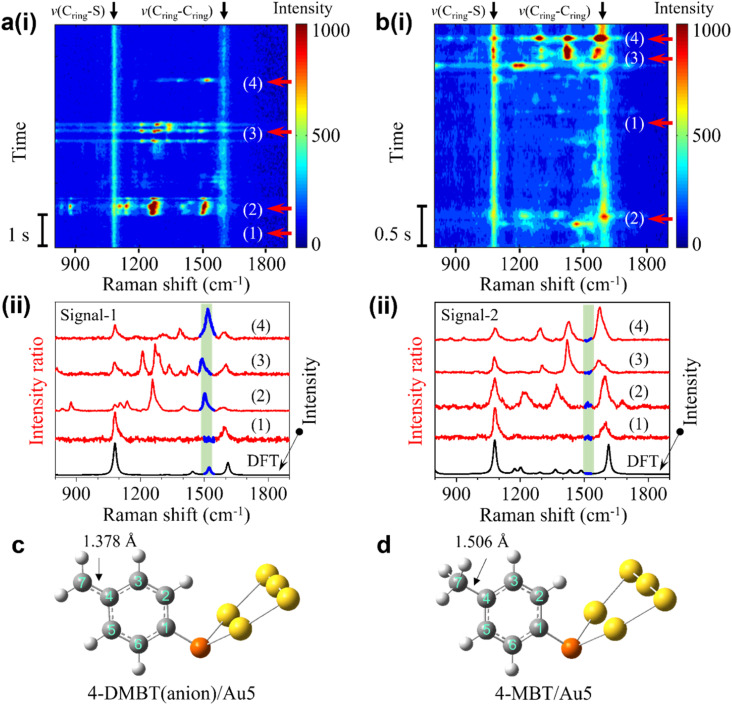
(a) (i) SERS time trajectory containing fluctuating signals near 1530 cm^−1^ (signal-1). (ii) Four stacked transient spectra from the time points in (a). (b) (i) SERS time trajectory of fluctuating signals without the peak near 1530 cm^−1^ (signal-2). (ii) Four stacked experimental spectra for on and off states from the time points in (c). (c) Calculated structure of 4-DMBT(anion)/Au_5_ for signal-1. (d) Calculated structure of 4-MBT/Au_5_ for signal-2.

The transient spectra containing the frequent bursts can be divided into two types, signal-1 with the 1530 cm^−1^ peak and signal-2 without this peak. The representative SERS time trajectories containing signal-1 and signal-2 are shown in Fig. 2a(i) and b(i), respectively. More examples are shown in S5 of the ESI.† Four transient spectra taken from different time points of the trajectories in row (i) are shown in row (ii) of Fig. 2a and b, respectively. The spectra at time point 1 marked in the heatmap in both trajectories are stable and do not involve a burst event and are shown for comparison. The transient spectra at time points 2, 3, and 4 from both trajectories are recorded during the intensity bursts. In Fig. 2a(ii), the peaks in the green shaded region are near 1530 cm^−1^ and can be easily identified. No corresponding peaks are observed in the same green shaded region in the transient spectra in Fig. 2b(ii).

We speculate that the appearance of the transient new peak in signal-1 is from the deprotonated 4-MBT (4-DMBT). The hot electrons excited by SP may cleave the C–H bond of the methyl group and generate 4-DMBT first in the benzyl radical form and then in the anion form (see Fig. 1a and S6†). We have calculated the Raman spectra of 4-MBT, the 4-DMBT radical and anion complexed with Au5 clusters (see Fig. 2a(ii), b(ii) and S7†). We found that a new vibration mode at 1523 cm^−1^ appears only in the 4-DMBT(anion)/Au_5_ structure. In the 4-DMBT(anion)/Au_5_ structure (Fig. 2c), the bond length between C_4_ and C_7_ of the benzyl anion is 1.378 Å, which is much shorter than the C_4_–C_7_ single bond (1.506 Å) of 4-MBT (Fig. 2d) but longer than a typical (non-benzene) C=C double bond (∼1.34 Å).^38^ We thus called it the unsaturated C=C bond (C_ring_=C_methylene_). Because of the resonance structure, the C_4_–C_7_ bond length is close to the C–C bond length (∼1.39 Å) of a benzene ring.^38^ For comparison, the bond length between C_4_ and C_7_ of the benzyl radical is slightly longer at 1.400 Å. Therefore, this new vibrational mode is attributed to the stretching of the C_4_=C_7_ bond of the 4-DMBT anion, which we denoted as *v*(C_ring_=C_methylene_) mode.

Moreover, the intensity bursts are also similar to the picocavity induced single-molecule spectral fluctuations, which is further confirmed by analyzing the spectra in the antistoke regions (Fig. S8 and S9†).^29,30,37,39^ The width of the broad peak near 1530 cm^−1^ in the average spectrum in Fig. 1c is directly related to the variations of the peak position of *v*(C_ring_=C_methylene_) mode in the transient spectra (see Fig. 2a(i)). We thus conducted LEFE calculation to understand how the spectral changes of *v*(C_ring_=C_methylene_) mode of the 4-DMBT anion are affected by the different location of the picocavity. We found that the *v*(C_ring_=C_methylene_) mode could be greatly enhanced when the picocavity is near the Au_2_ cluster (see Fig. S10 of the ESI†). Meanwhile, the relative positions of the interacting gold adatom and the methylene group also induce the spectral shift of the *v*(C_ring_=C_methylene_) mode. It is worth noting that due to the different positions of the gold adatoms, other peaks near 1310 cm^−1^, 1450 cm^−1^, *v*(C_ring_–S) and *v*(C_ring_–C_ring_) modes may also be enhanced with various degrees in signal-1. Interestingly, a peak near 800 cm^−1^ was previously observed due to the wagging vibration of the methylene group (both benzyl radical and anion) enhanced by the Ag–methylene interaction.^25,26^ However, we found that this peak shifted dramatically from 400 to near 1100 cm^−1^ when changing the Au–C (of methylene) distance from 5.5 to 2.0 Å in the structure of Au_5_/4-DMBT anion/Au_2_ (see Fig. S19†). Such Au–C distance changes are common in a stochastic picocavity. In contrast, under the same Au–C distance change, the peak shift range of the *v*(C_ring_=C_methylene_) mode is confined in the small range between 1500 and 1530 cm^−1^, and thus easier to recognize. Signal-2 is attributed to the transient 4-MBT signals due to the presence of picocavities (Fig. S10†). In signal-2, multiple peaks near 1200 cm^−1^, 1335 cm^−1^, 1440 cm^−1^, *v*(C_ring_–S) and *v*(C_ring_–C_ring_) modes may be enhanced to various degrees (see Fig. 2b(ii) and S10 of the ESI†).

Why the picocavity associated signals are only observed from the 4-MBT modified GNEs but not from the 4-TFMBT or BT modified GNEs? The picocavities formed by gold adatoms are often induced by molecule–metal interactions.^30,37^ However, the methyl group of 4-MBT, the –CF_3_ group of 4-TFMBT, and the benzene ring of BT all interact weakly with the surface gold atoms of GNPs. Therefore, we speculate that the benzyl carbanion of the dehydrogenation product may have a relatively strong interaction with the gold atoms on the GNP surface, greatly increasing the occurrence rate of observable picocavities. Indeed, from DFT calculations, the binding energy is only −6.9 kcal mol^−1^ for methyl–gold interaction but more than 5 times higher for carbanion–gold interactions (see Table S2 of the ESI†). Additionally, previous studies have also shown that the metal atoms or ions can form a stable bonding structure with the carbanion groups after dehydrogenation.^25,26,40,41^

**Fig. 3:**
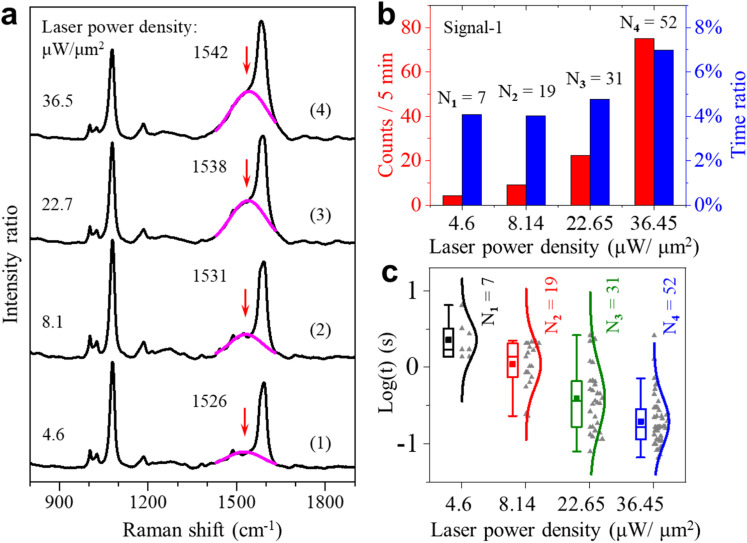
(a) Four representative averaged SERS spectra under four different laser power densities. The pink lines are the Gaussian fits to the broad peaks near 1531 cm^−1^. (b) Appearance event counts (red) and total appearance time percentage (blue) for signal-1 under different laser power densities. (c) Lifetime of signal-1 on the logarithmic scale under four different laser power densities.

If the dehydrogenation reaction is driven by plasmons, we expect that the occurrence rate of the spectral fluctuations depends on the power density of the irradiating laser. We then conducted laser power density dependence experiments. The typical results of intensity trajectories are shown in Fig. S11.† Indeed, the occurrence is much less at a lower power density of 4.6 μW μm^−2^ but significantly higher at 22.7 and 36.5 μW μm^−2^. At the higher laser intensities, more transient changes of the single-molecule signal appear, which often last for tenths of a second. Fig. 3a shows the averaged spectra at four different laser intensities from the four trajectories (104 s long) shown in Fig. S11.† The broad peak centered near 1530 cm^−1^ is obvious in all four spectra. Its peak height increased continuously with the increase of laser intensity from 4.6 to 36.5 μW μm^−2^. This height mainly reflects the intensity and occurrence rate of the transient peaks in the green shaded region near 1530 cm^−1^ in Fig. 2a(i). The higher laser intensity leads to the production of more 4-DMBT and picocavities, which results in an increase of the broad peak height. Interestingly, the broad peak position also gradually shifted to a higher wavenumber with the increase of the laser intensity. With the increase of laser power density, the gold adatoms on the GNP surface become more active with a shorter lifetime, so the interactions between gold adatoms and the methylene groups are less stable. This weakens the Au–methylene interaction, which leads to the strengthening of the C_ring_=C_methylene_ bond of 4-DMBT and thus the blue shift of the *v*(C_ring_=C_methylene_) peak.

We also carried out statistical analysis for the occurrence and the lifetime of the two types of signals under different excitation laser intensities. Fig. 3b shows the statistical results of the fluctuating events about signal-1 for the total-time percentage and frequency of occurrence under different laser power densities. From the results, we found that the occurrence of these fluctuations is proportional to the laser power densities. There is a sudden increase for signal-1 when the laser power density is increased to 36.5 μW μm^−2^. This observation indicates that the activation of the methyl group is affected to a large extent by the intensity of incident light. The continuous increase in occurrence can further indicate that the higher laser power density can make the dehydrogenation of the methyl group more frequent and faster. Fig. 3c shows the lifetime of fluctuating events with the peak of the *v*(C_ring_=C_methylene_) mode. From the results, we found that signal-1 is more frequent and short-lived at the higher power densities. The corresponding statistical results of the signal-2 events are shown in Fig. S12 of the ESI.†

To further understand the reaction mechanism, we calculated the Gibbs free energies of the possible reactions (see Fig. S6 of the ESI†). The C–H cleavage energy to generate a 4-DMBT radical (2.63 eV) is much lower than the energy required to generate an anion (8.84 eV). Therefore, we propose that a 4-DMBT radical is first formed as an intermediate when a hot electron transfers energy to break a hydrogen atom (hydrogen radical) from the methyl group (see Fig. 1a). In the next step, the highly reactive 4-DMBT radical can be converted to an anion by acquiring an electron, *i.e.*, from another hot electron. The resonance stabilized 4-DMBT anions can be further stabilized by interacting with the gold adatoms. Based on the DFT calculations (see Fig. S6 of the ESI†), the energy required for the dehydrogenation reaction is lower in an aqueous solution than in the air, which is attributed to the solvation effect and the large dielectric constant of water. We have compared the SERS results of the same GNE both in the air and aqueous solutions (see Fig. S13 of the ESI†). Interestingly, the dynamically fluctuating burst events disappeared when the GNE was in the air but reappeared when the GNE was placed in an aqueous solution. In addition, we also did not observe the burst events in pure ethanol (see Fig. S13 of the ESI†). Therefore, the aqueous solution is critical for the dehydrogenation reaction of 4-MBT. Because the dehydrogenation reaction is an oxidation reaction, we also studied the influence of dissolved oxygen molecules. The occurrence of signal-1 per 10 min dropped 20–30% after oxygen removal by Argon gas bubbling. Therefore, the presence of dissolved oxygen molecules can lower the reaction barrier. For example, the oxygen molecule may interact directly with the methyl group to accept the plasmon cleaved hydrogen atom (see Fig. S22†). However, without oxygen molecules in solution, the dehydrogenation reaction can still proceed effectively.

### The dimerization reaction

**Fig. 4:**
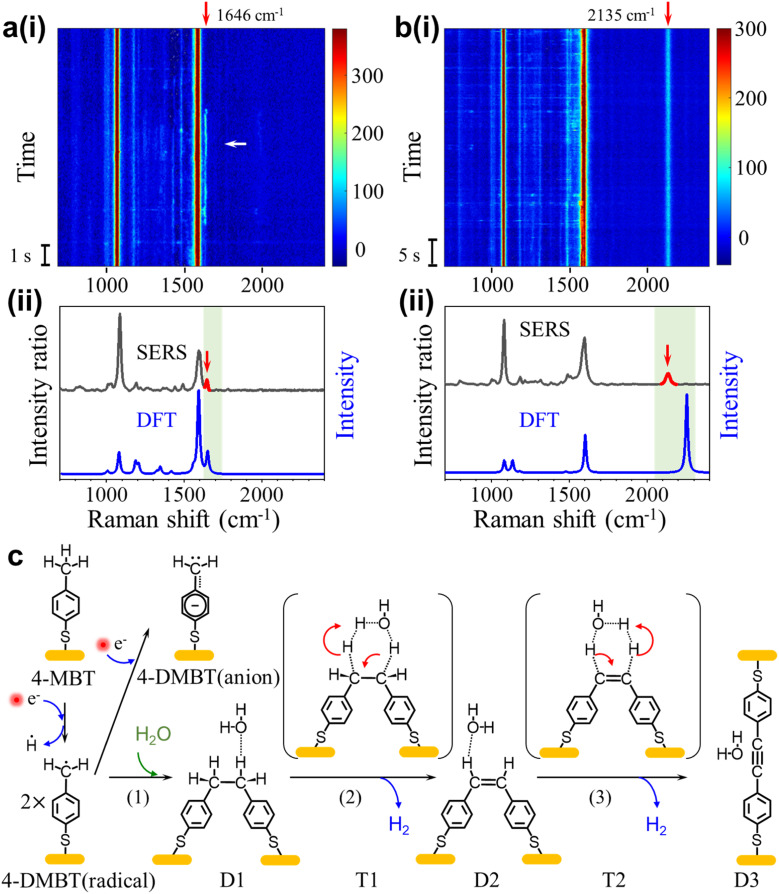
(a) (i) Representative SERS time trajectory of signal-3 with a transient peak near 1646 cm^−1^. (ii) Average spectra over 1 s at 52 s (indicated by the white arrow) in (a) and the DFT calculated Raman spectrum of the D2 structure. (b) (i) Representative SERS time trajectory of signal-4 with a stable peak near 2135 cm^−1^. (ii) Average spectra (over 1 s) at 20 s and the DFT calculated spectrum of the D3 structure. (c) A proposed reaction pathway from 4-MBT to D3 through the dimerization of the 4-DMBT radicals. T1 and T2 are the transition states of reactions (2) and (3), respectively. For DFT calculations, we ignored the Au connected to the thiol group to simply the calculations.

We also observed signals containing peaks near 1646 cm^−1^ and 2135 cm^−1^ that we referred to as signal-3 and signal-4, respectively. Signal-3 appears more often than signal-4, but both signals only occur occasionally compared to signal-1. The peak near 1646 cm^−1^ (signal-3) usually fluctuates with a lifetime of a few seconds and the peak near 2135 cm^−1^ (signal-4) is usually stable. Fig. 4a(i) shows the SERS heatmap of a representative signal-3 appearing for 6s. The average spectrum of signal-3 is shown in Fig. 4a(ii). Fig. 4b(i) shows the SERS heatmap of a representative signal-4 with the stable 2135 cm^−1^ peak. The average spectrum of signal-4 is shown in Fig. 4b(ii).

According to previous reports,^24,42^ the benzyl radicals can be dimerized. Through the DFT chemical reaction calculation, we proposed the reaction path from 4-MBT to dimers D1, D2 and D3, as shown in Fig. 4c. Driven by plasmons and assisted by the water and oxygen molecules in solution, 4-MBT is first dehydrogenated to form the benzyl radical (4-DMBT radical). The benzyl radicals can be quickly converted to benzyl anions (4-DMBT anions), which are stabilized by interacting with gold adatoms and can be detected by SERS. A fraction of the benzyl radicals can also form dimer D1, which is difficult to recognize from the SERS spectra due to the lack of signature peaks. Catalyzed by a hot electron and assisted by the water molecule (see the transition state T1), D1 loses two hydrogen atoms (radicals) to form D2 with a C=C bond and releases a hydrogen molecule. The hot electron further catalyzes the reaction from D2 to D3 through transition state T2, after losing two hydrogen atoms to release a hydrogen molecule. For both steps 2 and 3, the release of two neutral hydrogen atoms (and a hydrogen molecule) is preferred over the release of two protons (or water solvated protons), which involves charge separation and rebalance. The calculated energies for the intermediates and transition states can be seen in Fig. S18.†Fig. 4a(ii) and b(ii) also show the calculated spectra of D2 and D3, respectively. The signature peak near 1646 cm^−1^ of signal-3 is assigned to the *v*(C=C) mode of D2 and the signature peak near 2135 cm^−1^ of signal-4 is assigned to the *v*(C≡C) mode of D3. We did not capture the direct transition from D2 to D3 in real time. One reason is due to the overall low occurrence rate for signal-3 and signal-4. Also, the D2 is relatively stable and the lifetime of signal-3 (from D2) is often limited by the lifetime of picocavities. Nevertheless, the observation of dimers D2 and D3 also confirms the existence of a benzyl radical as a key intermediate after methyl dehydrogenation.

### The effect of electrode potential on the dehydrogenation reaction

**Fig. 5:**
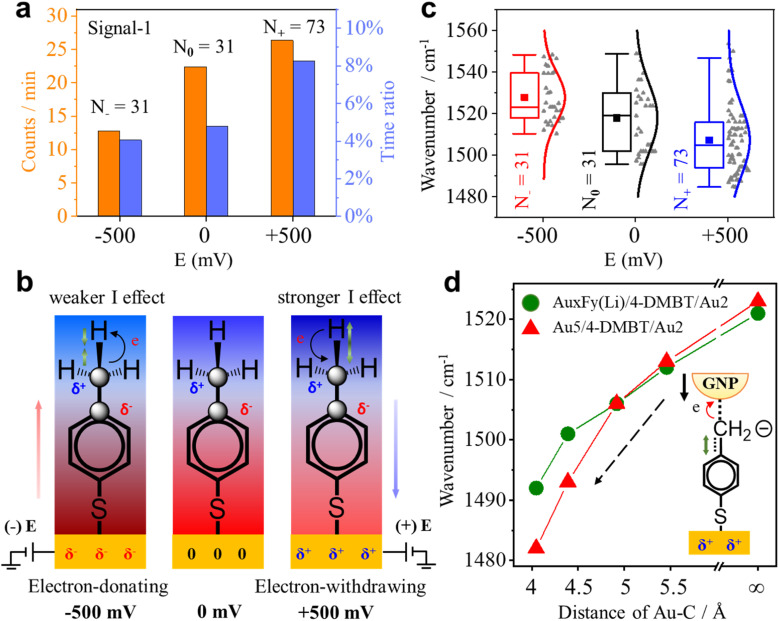
(a) Occurrence rate (orange column) and appearance time ratio (blue column) under different *E*_s_. (b) Illustrations of the change in the strength of the I-effect and in the bond length of the associated chemical bonds. The charge distribution for 4-MBT under different *E*_s_ is also shown. The blue shades represent the positive group and the red represents the negative group. From left to right, the electronegativity of the phenyl group decreases. The green arrow indicates the change in the C–H bond length. The black arrow shows the direction of the electron cloud shift. (c) Box plots for the position of the peak near 1530 cm^−1^ of signal-1 under different *E*_s_ with the raw data (gray dots) in scatter plots. The solid lines are the Gaussian fits. The solid square dots represent the mean values of the wavenumber. *N* is the total number of events under each *E*. (d) Plots of the *v*(C_ring_=C_methylene_) peak position of the 4-DMBT anion as a function of the interaction distances (Au–C) between the Au_2_ cluster and methylene group determined by using DFT calculation. The red triangles are for the structure of Au_5_/4-DMBT(anion)/Au_2_ and the green solid dots are for the structure of Au_*x*_F_*y*_(Li)/4-DMBT(anion)/Au_2_ in which the partial gold atoms in Au_5_ are replaced by F or Li atoms.

Since the benzene ring is more electronegative than the methyl group, the C–C σ-bond of 4-MBT has an obvious inductive effect (I-effect) with the tendency of electron transfer from the methyl to the phenyl group. The I-effect weakens the C–H bond of the methyl group and thus makes the dehydrogenation reaction easier (see Fig. S14 of the ESI†). As demonstrated by recent studies,^32,43^ the electrode can polarize the attached molecules and generate the electro-inductive effect. We are interested to know if the electrode potential (*E*) applied to the GNE can modulate the I-effect and consequently the dehydrogenation reaction of 4-MBT. The GNEs were subjected to +500, zero and −500 mV of *E versus* the Ag/AgCl wire quasi-reference electrode. As shown in Fig. 5a, the occurrence rate (counts per min) of signal-1 is more than doubled at +500 mV compared to at −500 mV. We also derived the appearance time ratio at each *E* by dividing the total duration of all transient signal-1 events by the total measurement time. The same as the occurrence rate, the appearance time ratio is higher at +500 mV.

To understand how the applied *E* modulates the I-effect of 4-MBT, we calculated the charge distribution of various Au cluster-4-MBT complex structures at both positive and negative *E*_s_ (see Fig. S15 and Table S3 in the ESI†). Fig. 5b shows the diagrams of electron shift under different *E*_s_ according to the calculated results. With a positive *E* on the GNE, the GNE becomes an electron-withdrawing group and works together with the benzene ring to lower the electron density of the methyl group and thus strengthen the I-effect. So, the methyl group is more reactive. Conversely, the negative *E* on the GNE makes the GNE an electron-donating group and weakens the I-effect. Thus, the methyl group is less reactive (see Section S17 of the ESI†).

Interestingly, the applied *E* affects not only the dehydrogenation reaction rate of the methyl group but also the signature peak position of the 4-DMBT anion. Fig. 5c shows the peak position distributions of the *v*(C_ring_=C_methylene_) mode in signal-1 at different *E*_s_. The mean wavenumbers of the *v*(C_ring_=C_methylene_) mode peak at −500, zero and +500 mV are 1528 cm^−1^, 1518 cm^−1^ and 1507 cm^−1^, respectively. The decrease of the peak position (red shift) at the positive *E* suggests the weakening of the C_ring_=C_methylene_ bond. Therefore, the applied *E* also alters the strength of the C_ring_=C_methylene_ bond of the 4-DMBT anion. The applied *E* not only induces the electron cloud shift but also induces the change of the nanogap distance of NPoNE structures. For the latter, the applied *E* can change the nanogap distance because of the electrostatic force between the GNE and the negatively charged GNPs (see Fig. S16 of the ESI†).^35,36^ The positive (or negative) *E* reduces (or increases) the nanogap distance because of the attractive (repulsive) force. To understand the roles of both effects in the peak position change of *v*(C_ring_=C_methylene_) mode, we carried out DFT calculations for various Au_*x*_F_*y*_(Li)/4-DMBT(anion)/Au_2_ and Au_5_/4-DMBT(anion)/Au_2_ structures with different Au–C distances (see Fig. S17 of the ESI†). Because the F (fluorine) atom has a strong electron-withdrawing ability and the Li (lithium) atom has a strong electron-donating ability, we replace Au with F atoms in the Au_5_ cluster to simulate the electron shift at positive *E* and replace Au with Li atoms to simulate the electron shift at negative *E*. The results are shown in Fig. 5d. With the decrease of the Au–C distance, reflecting the change of *E* from negative to positive, the wavenumber of the *v*(C_ring_=C_methylene_) peak of the Au_*x*_F_*y*_(Li)/4-DMBT(anion)/Au_2_ structure becomes smaller. The trend is consistent with the experimental results in Fig. 5c. The same trend is also observed for the structure of Au_5_/4-DMBT(anion)/Au_2_ but the curve is even steeper. Therefore, the *E*-induced nanogap distance change plays a dominant role in affecting the peak position of *v*(C_ring_=C_methylene_) mode. The nanogap distance change can effectively modulate the interaction between the gold and benzyl carbanion (see Section S12 of the ESI†), thus affecting the strength of the C_ring_=C_methylene_ bond.

## Conclusion

In summary, we have used time-resolved SERS measurements to probe the plasmon-mediated C–H bond activation of the aromatic methyl group on the apex of GNEs *in situ* and in real-time in neutral aqueous solution at room temperature. Significant fluctuations of SERS signals have been observed for 4-MBT, but not for 4-TFMBT. The origin of these fluctuating spectral changes has been revealed based on DFT calculations and the picocavity model. The surface plasmon decay-induced hot electrons and local heat activate the dehydrogenation reaction of 4-MBT, leading to the formation of a benzyl radical, aided by the presence of water and oxygen. The short-lived benzyl radical is then converted to a benzyl carbanion. The stronger interaction between the benzyl carbanion and the surface gold atoms of GNPs generates gold adatoms, which stabilizes the benzyl carbanion and strongly enhances the SERS signals through the picocavity effect. We also demonstrated that the benzyl radical intermediates from the plasmon-mediated dehydrogenation reaction can undergo self-reactions and form dimers under mild conditions, as revealed by the signature SERS peak of carbon–carbon triple bonds. We further applied positive electrode potentials to the GNE to effectively enhance the inductive effect, thus enhancing the reaction rate of the dehydrogenation reaction. This would be an important and facile means to increase the yield of the reaction. Therefore, the PMCR can indeed provide a green and facile reaction mechanism for the selective activation of the aromatic methyl groups to produce important benzyl radicals, thus enabling a wide range of subsequent reactions.

## Data availability

The characterization of the GNE, background correction, additional data, control experiments, detailed DFT calculations, picocavities, and the local electric field enhancement calculations have been uploaded as part of the ESI.†

## Author contributions

J. H. Z.: investigation, data curation, formal analysis, DFT calculations, and writing – original draft. J. G.: methodology and software. G. G.: SEM, validation, and writing – review & editing. A. M. M.: supervision, software, and validation. S. C.: supervision, software, and resources. J. H.: conceptualization, supervision, resources, and writing – original draft & editing.

## Conflicts of interest

The authors declare no competing financial interest.

## Supplementary Material

SC-014-D3SC05847F-s001
