# Supplementary material for: Plasmon-mediated dehydrogenation of the aromatic methyl group and benzyl radical formation
Source: Chem Sci. 2023 Nov 28;14(47):13951–61. doi: 10.1039/d3sc05847f (PMC10699572; doi:10.1039/d3sc05847f)
Supplement: SC-014-D3SC05847F-s001 [file SC-014-D3SC05847F-s001.pdf]

Electronic Supplementary Material (ESI) for Chemical Science.
This journal is © The Royal Society of Chemistry 2023

Electronic Supplemental Information
for
Plasmon-Mediated Dehydrogenation of Aromatic Methyl Group and Benzyl Radical Formation

Jianghao Zhou[a,b], Jing Guo[b], Govinda Ghimire[b], Alexander Mebel[c], Shuai Chang*[a] and Jin He*[b,d]

[a] The State Key Laboratory of Refractories and Metallurgy, School of Materials and Metallurgy, Wuhan University of Science and Technology, Wuhan, 430081, China.

[b] Department of Physics, Florida International University, Miami, Florida 33199, USA.

[c] Department of Chemistry and Biochemistry, Florida International University, Miami, Florida 33199, USA.

[d] Biomolecular Science Institute, Florida International University, Miami, Florida 33199, USA

Corresponding Authors

*E-mail: schang23@wust.edu.cn.

*E-mail: jinhe@fiu.edu.

Table of Content

1. The Raman spectra of 4-MBT powder and the calculated Raman spectra of 4-MBT 3

2. GNE fabrication, surface modification, and characterization 3

3. SERS spectra background correction 5

4. Control experiments with BT molecules 6

5. Extra data for the 4-MBT and 4-DMBT signals 6

6. DFT calculations 7

7. The deprotonation and dehydrogenation reactions of 4-MBT 8

8. The origin of signal-1 9

9. Vibration mode assignments 9

10. The confirmation of picocavity origin by stoke/antistoke measurements 11

11. The local electric field enhancement calculation for 4-MBT and the 4-DMBT anion 12

12. The interaction of methyl-gold and methylene-gold 14

13. The representative SERS signals under different laser power densities 15

14. The statistical results of the fluctuating signal-2 events under different laser intensities 16

15. Control experiments in the air and ethanol solution 16

16. The inductive effect lowers the cleavage energy for the 4-MBT dehydrogenation reaction 18

17. Electrode potential (E) modulation for 4-MBT SERS signals 18

18. The evidence of nanogap distance modulation by electrode potential (E) 20

19. The effects of applied E and nanogap distance for 4-DMBT anion signals 21

20. The calculated energies in the dimerization reaction 23

21. The wagging vibration mode of methylene group in 4-DMBT 24

22. The influence of oxygen on 4-MBT dehydrogenation 25

23. DFT calculation of 4-MBT dehydrogenation with the involvement of oxygen 27

1. The Raman spectra of 4-MBT powder and the calculated Raman spectra of 4-MBT

As shown in Fig. S1, the calculated spectrum matches reasonably well can well with the measured spectrum of powder. The details of DFT method are given in section S6. The assignments for the main vibration modes are listed in Table S1.

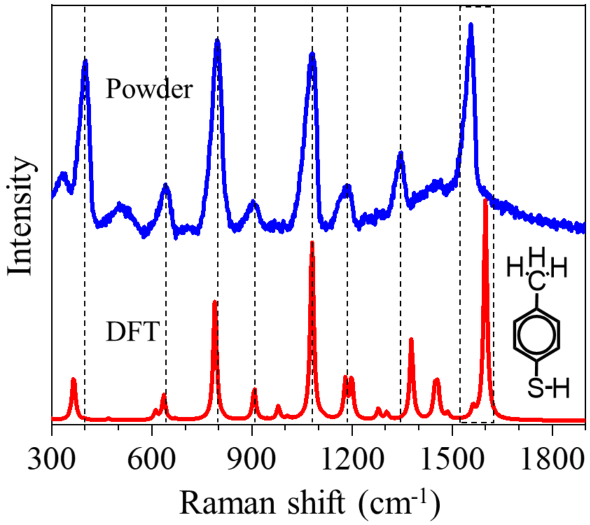

Fig. S1. An experimental spectrum of 4-MBT powder (blue) and the calculated Raman spectrum of 4-MBT molecule (red).

2. GNE fabrication, surface modification, and characterization

We have optimized the GNE size, shape, and surface modification parameters to achieve the best signals in the SERS measurements. The details of the general GNE fabrication have been described elsewhere.[1, 2] In brief, the GNE was prepared from a 0.2 mm diameter gold wire (Electron Microscopy Sciences, 99.99%) *via* an electrochemical etching method. After cleaning, the GNE was partially insulated with a high-density polyethylene (HDPE) coating. After HDPE insulation, the GNE was electrochemically cleaned in 0.5 M H_2SO_4 solution using a potentiostat (CHI760D, CH Instruments, Inc., USA). The exposed surface area of the GNE apex was estimated based on the diffusion-limited current in the steady state cyclic voltammetry (CV). The typical diffusion-limited current is about 60 nA and the effective exposed GNE apex surface is approximately 12 μm^2.

For chemical modification, the insulated GNE was immersed in the molecule solution overnight (12 h) at room temperature. The 10 mM 4-MBT solution was prepared by dissolving 4-MBT

powders in absolute ethanol with sonication for 10 mins. Then the GNE was sequentially rinsed with 70% alcohol and DI water. We have used CV to evaluate the quality and surface coverage of 4-MBT modification. As is shown in Fig S2a, we have observed about 39% current decrease in the diffusion-limited current (i_d) after the 2h 4-MBT modification. Based on the previous work,[1, 3] there was about 83% current decrease for the benzenethiol(BT) modified GNE. The typical surface coverage of the BT molecule is about 1.4 per nm^2 (0.23 nmol/cm^{-2}) on a flat Au (111) substrate. Here, we estimated the surface coverage of the 4-MBT on the curved GNE apex was over half of that of BT and thus we used 0.8 per nm^2 for 4-MBT.

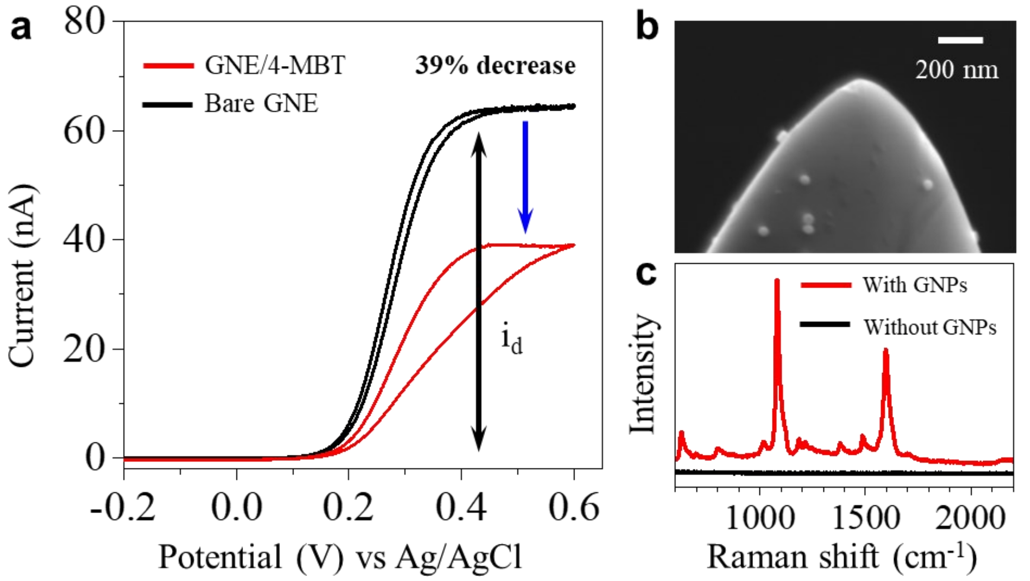

Fig. S2. (a) CVs of a GNE before (black color curve) and after 4-MBT (red color curve) modification for 2 h. The CVs were carried out in 1 M KCl solution (pH = 7.0) with 0.1 M Potassium Ferricyanide solution. Scan rate: 50 mV·s^{-1}. (b) Another SEM image for a 4-MBT modified GNE apex with absorbed GNPs. (c) The SERS spectra from a 4-MBT modified GNE before (red spectrum) and after (black spectrum) GNP adsorption.

We did a crude estimation for the number of molecules that contributed to the major SERS signals in the measurements. Based on previous FDTD simulation,[4] the diameter of the hotspot (the region of 10^9 electromagnetic enhancement) between the GNE and the 40 nm GNP (with a gap distance of 0.91 nm, close to the 4-MBT length) is about 3.8 nm and thus the cross-section area of the hotspot is about 11 nm^2. Therefore, there are about 8.8 molecules in each hotspot of a NPoNE

structure. From the SEM images (both Fig. 1b and Fig. S2b), there are about 4~5 GNPs per μm^2 on the GNE apex. Few GNPs adsorbed on GNE are caused by the weak interaction between GNPs and 4-MBT. The NPoNE structures can greatly boost the SERS signals. Without the adsorption of GNPs to the GNE apex, the SERS signal is too weak to be detected at the same condition (see Fig. S2c). In the experiment, the laser beam size (about 6 μm in diameter) is bigger than the apex area. If the surface area of GNE apex covered by the laser beam is about 6 μm^2, there are about 264 molecules in the hotspots during the measurement. This estimation has not considered the picocavity situation. Due to the existence of picocavities in the hotspot, we can detect SERS signals from a single molecule and even single bond changes.

3. SERS spectra background correction

The backgrounds of the collected SERS spectra were fitted by the asymmetric least square's method. For the stable SERS trajectories, we subtracted the average background acquired at the stable region. For the highly dynamic SERS trajectories, we subtracted the baseline acquired at the stable region. To correct the plasmon background effect of individual spectra, the background subtracted SERS spectra were further divided by the background accordingly.[5] One example is shown in Fig. S3. After background division, the intensity is changed to the intensity ratio.

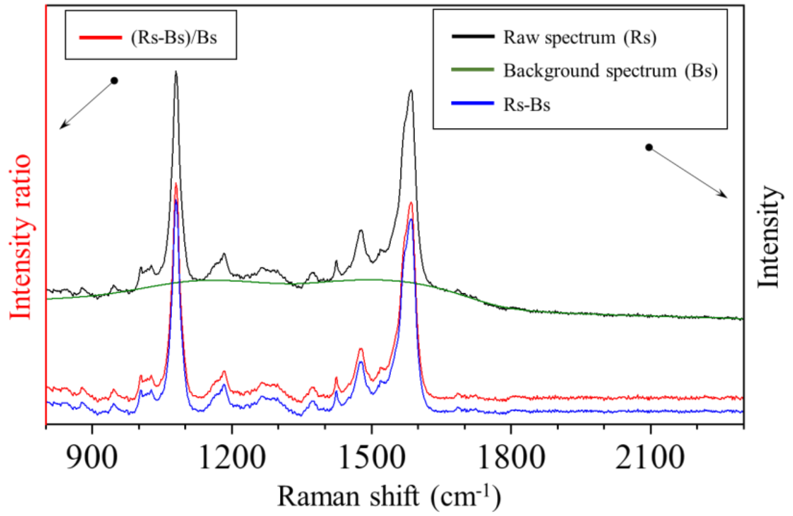

Fig. S3. Original SERS spectrum (Rs, black), the fitted background (Bs, green), the background subtracted SERS spectrum (Rs-Bs, blue) and the background subtracted SERS spectra divided by the background ((Rs-Bs)/Bs, red).

4. Control experiments with BT molecules

We have also modified the GNP surface with benzenethiol (BT) molecules to form GNE/BT/GNP junctions. Fig. S4 show the representative SERS time trajectory. We did not observe any ‘blinking’ signals. In Fig. S4 b, the averaged spectrum (red) matches well with the calculated spectrum (black) using the Au5/BT structure shown at the right ride.

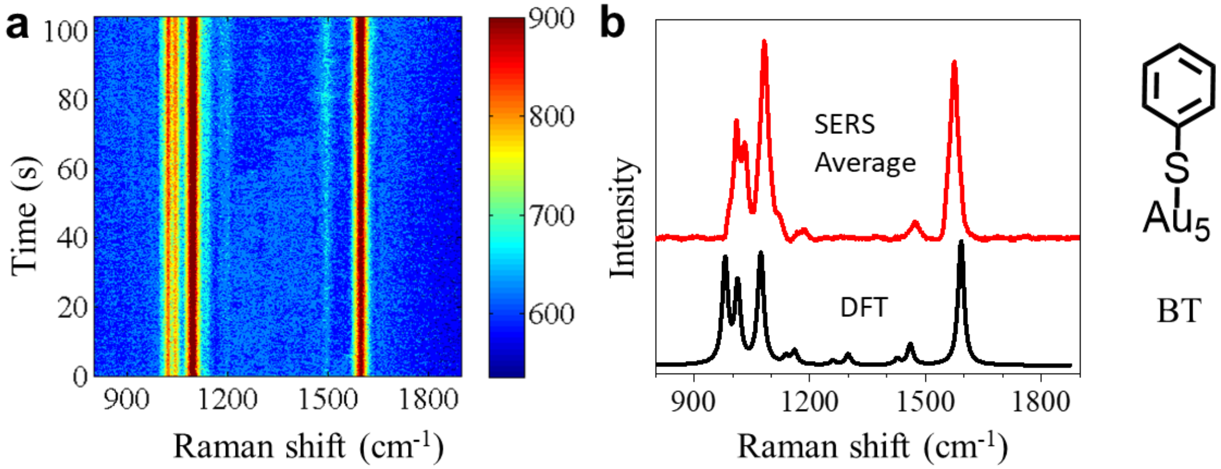

Fig. S4. Control experiments for BT molecule. (a) Representative SERS trajectory in the intensity heatmap format. (b) Average spectrum (red) from the heatmap in (a) and the DFT calculated spectrum of BT (black) based on the structure at the right ride.

5. Extra data for the 4-MBT and 4-DMBT signals

Fig. S5 shows more SERS time trajectories containing transient signals of 4-DMBT (signal-1) and 4-MBT (signal-2).

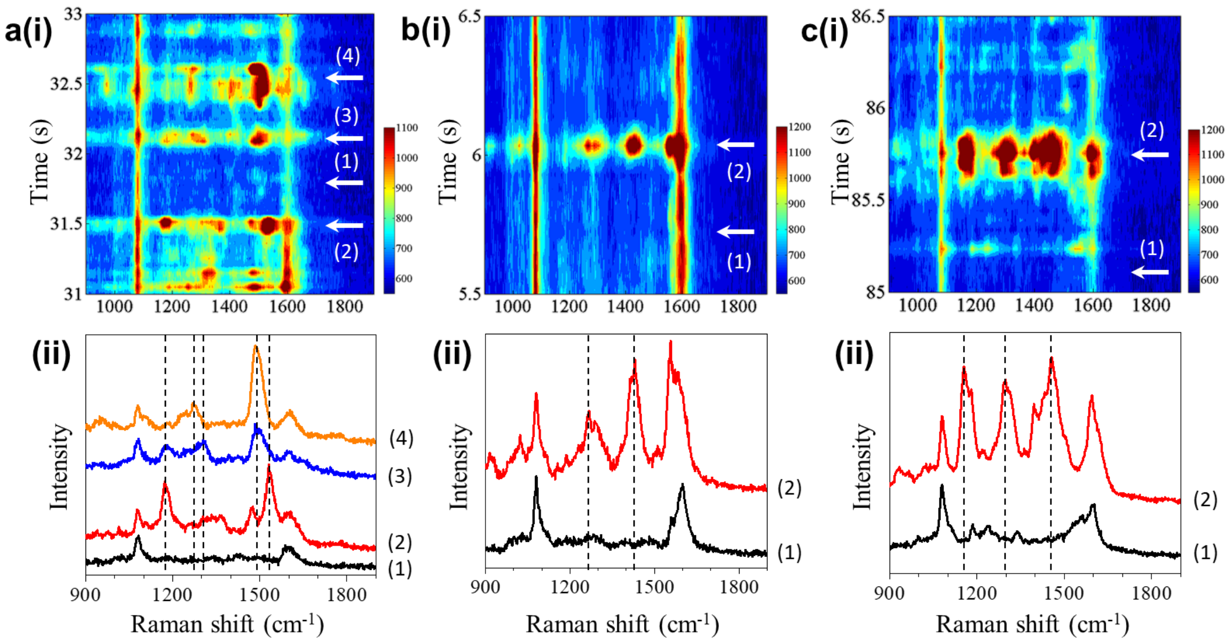

Fig. S5. (a) SERS time trajectory (i) containing signal-1 for 4-DMBT. (b-c) SERS time trajectories (i) containing signal-2 for 4-MBT. The corresponding stacked spectra (ii) at different time points are shown in (ii). The black color spectra (1) are selected from the 'off' state.

6. DFT calculations

We have constructed Au cluster-molecule complex structures in Gaussian software to calculate the Raman spectra and charge distributions. We often use the Au_5 cluster to simulate GNE and Au_2 cluster to simulate GNP. The DFT calculations have helped us to gain atomic-level insight into the molecular-gold interaction and the possible structures of the plasmonic molecular junctions. We used the wB97XD functional to optimize these structures at the 6-311g (d, p) level for C, H, S, Li, and F atoms and the MWB60 level for the Au atom. The wB97XD uses DFT-D2 dispersion correction to improve the accuracy of weak interaction calculation, which is beneficial for the calculation of electron transfer. The calculated Raman spectra are plotted by applying a Lorentzian function with a full-width at half-height (FWHH) of 10 cm^{-1}. To match the calculated spectra with the measured spectra, we use the $\nu(C_{ring}\text{-}S)$ peak position as the reference to determine the scaling factor. The scaling factor is 0.9643 for neutral molecules such as 4-MBT, 4-TFMBT, and BT. For both 4-DMBT anion and radical, the scaling factor is 0.9845. Based on the optimized

structures, we also carried out the LEFE calculation for Au_5/4-MBT/Au_2 and Au_5/4-DMBT/Au_2 structures by placing the hot spot at different positions.

7. The deprotonation and dehydrogenation reactions of 4-MBT

We calculated the Gibbs free energies (ΔG) for the dehydrogenation reactions of 4-MBT to produce 4-DMBT in both radical and anion forms both in the water solvent and in the air. The results are shown in Fig. S6. The calculated ΔGs are always higher in the air than in the water solution. Here, we focus on the results calculated in an aqueous solution. We found that the hydrogen atom (radical) cleavage energy from 4-MBT to its radical form (2.63 eV) are much lower than the proton cleavage energy to its anion form (8.84 eV). Therefore, the C-H bond of 4-MBT is most likely first cleaved by the high-energy hot electron to produce a 4-DMBT radical and a hydrogen atom. Then, the 4-DMBT radical acquires an electron (*i.e.*, through the electron transfer from a hot electron) to become the 4-DMBT anion. Notably, the anionic form is more stable than the radical form. The hydrogen radicals, that are produced during cleavage, usually move away through binding with water molecules.

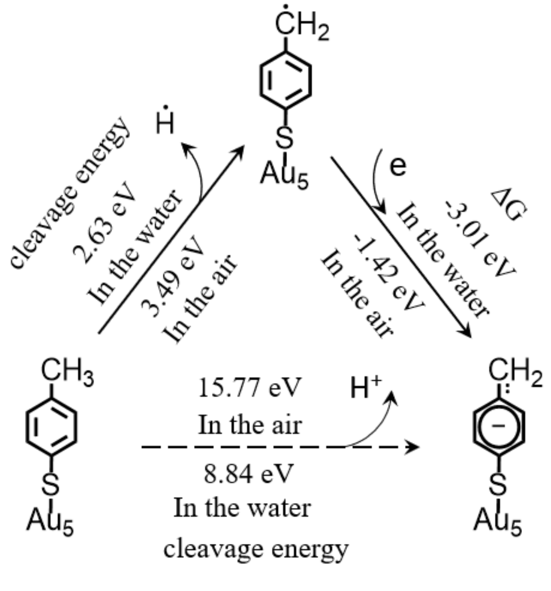

Fig. S6. The 4-MBT dehydrogenation/deprotonation reaction pathway (with energy) to generate radical/anion forms of 4-DMBT.

8. The origin of signal-1

To identify the source of signal-1, we calculated the Raman spectra of the radical and anion of 4-DMBT. Fig. S7 shows the spectra of two structures calculated by DFT which are Au5/4-DMBT radical and Au5/4-DMBT anion, respectively. We found that only the spectrum of 4-DMBT anion has a characteristic peak at 1523 cm^{-1}, while there is no such peak in the spectrum of 4-DMBT radical. Therefore, we determine that signal-1 is mainly from the anionic form of 4-DMBT.

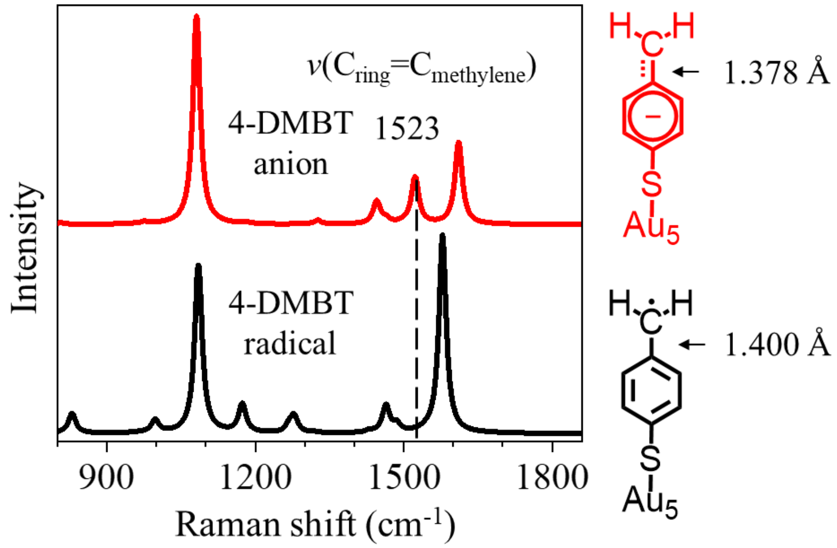

Fig. S7. The DFT calculated spectra of Au5/4-DMBT radical and Au5/4-DMBT anion. The corresponding molecular structures are shown at the right ride.

9. Vibration mode assignments

Based on the DFT calculations, we listed the major vibration modes for 4-MBT, Au5/4-MBT, Au5/4-MBT/Au2, 4-DMBT(anion), Au5/4-DMBT(anion), and Au5/4-DMBT(anion)/Au2 structures in the spectral range between 1000 cm^{-1} and 1800 cm^{-1} in Table S1 below. The experimental results are the mean values within the range of fluctuation. The signature peaks are marked in bold.

Table S1. The major vibration modes of the six structures

Structure	Experimental frequency (cm^{-1})	DFT frequency (cm^{-1})	Assignment[a]
4-MBT	1556	1599	ring stretching ($\nu(C_{ring}–C_{ring})$)

	**1346**	**1378**	**ν CH_3**
	**1183**	**1180**	**ν C_{ring}-C_{methyl}** + ρ C_{ring}H
	1080	1080	in plane ring breathing+ν(C_{ring}-S)
Au5/4-MBT	-	1606	ring stretching (ν(C_{ring}–C_{ring}))
	**-**	**1366**	**ν CH_3**
	**-**	**1195**	**ν C_{ring}-C_{methyl}** + ρ C_{ring}H
	-	1074	in plane ring breathing+ν(C_{ring}-S)
Au5/4-MBT/Au2	1600	1615	ring stretching (ν(C_{ring}–C_{ring}))
	**1370**	**1335**	**ν CH_3**
	**1220**	**1202**	**ν C_{ring}-C_{methyl}** + ρ C_{ring}H
	1080	1081	in plane ring breathing+ν(C_{ring}-S)
4-DMBT(anion)	-	1595	ring stretching (ν(C_{ring}–C_{ring}))
	-	**1504**	**ν C_{ring}≈$C_{methylene}$ + ρ CH_2** + ρ C_{ring}H
	-	**1443**	**ρ CH_2** + ρ C_{ring}H
	-	**1318**	**ρ CH_2** + ν C_{ring}≈$C_{methylene}$ + ρ C_{ring}H
	-	1067	in plane ring breathing+ν(C_{ring}-S)
Au5/4-DMBT(anion)	-	1603	ring stretching (ν(C_{ring}–C_{ring}))
	**-**	**1516**	**ν C_{ring}≈$C_{methylene}$ + ρ CH_2** + ρ C_{ring}H
	**-**	**1440**	**ρ CH_2** + ρ C_{ring}H
	**-**	**1320**	**ρ CH_2** + ν C_{ring}≈$C_{methylene}$ + ρ C_{ring}H
	-	1076	in plane ring breathing+ν(C_{ring}-S)
Au5/4-DMBT(anion)/Au2	1596	1605	ring stretching (ν(C_{ring}–C_{ring}))
	**1530**	**1506**	**ν C_{ring}≈$C_{methylene}$ + ρ CH_2** + ρ C_{ring}H
	**1425**	**1450**	**ρ CH_2** + ρ C_{ring}H
	**1270**	**1314**	**ρ CH_2** + ν C_{ring}≈$C_{methylene}$ + ρ C_{ring}H
	1080	1086	in plane ring breathing+ν(C_{ring}-S)

[a] The notations of the modes are: ν = stretch, σ = scissor, δ = deformation, ρ = rock, and β = bend.

10. The confirmation of picocavity origin by stoke/antistoke measurements

Previous studies have also found picocavities in the gold nanogap.[6] The presence of picocavity makes the peak value significantly higher in antistoke signal.[7] To confirm the picocavity origin of signal-1, we measured the anti-stoke spectra. Fig. S8 shows a representative SERS time trajectory with signal-1 for both anti-stoke (a) and stoke (b). The signature peak of signal-1 near 1515 cm^{-1} is pronounced in both stoke and antistoke spectra and is especially strong in the antistoke spectra than other peaks. Based on the DFT calculation, this new peak is from the 4-DMBT structure. The appearance and selective enhancement of this new peak in both stoke and anti-stoke spectra indicate that 4-MBT undergoes a dehydrogenation reaction while its characteristic peak is enhanced by the picocavity.

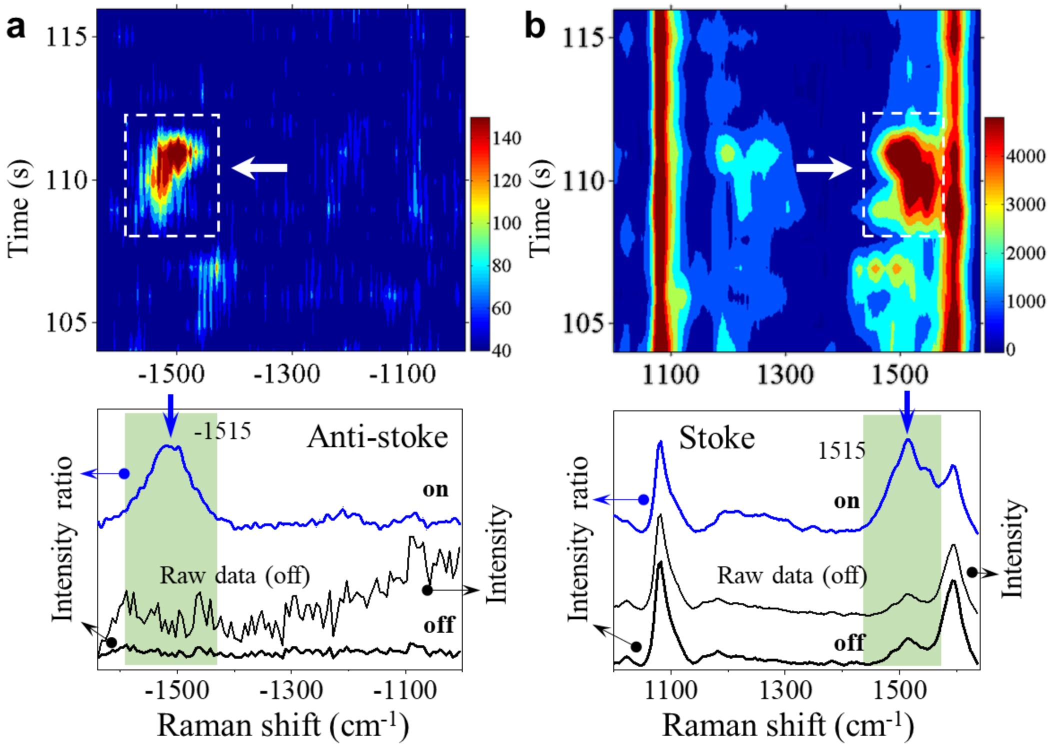

Fig. S8. (a-b) Representative SERS time trajectories containing signal-1 with a peak near 1500 cm^{-1} for anti-stoke (a) and stoke (b) spectra. The exposure time is set to be 1 s. Stacked transient spectra (after baseline correction as described in section s3, with intensity ratio y axis at the left side) at "on" and "off" states taken from the heatmaps are shown at the bottom. For comparison, the raw spectrum at the "off" state before baseline correction is also shown.

We not only observed an obvious peak of signal-1 in antistoke region but also observe the distinct peaks of a typical signal-2 in antistoke. Fig. S9 shows the SERS heatmap (above) and spectrum (below) of signal-2 in antistoke (a) and stoke (b). We select the spectrum from on-state (at 53~54 s) and off-state (at 55~57 s). We found the obvious peak at -1421 cm^{-1} (in antistoke) and 1421 cm^{-1} (in stoke). In addition, we found a small peak occurring at -1191 cm^{-1} (in antistoke) and 1191 cm^{-1} (in stoke). According to the DFT calculation, the peak at 1191 cm^{-1} is assigned to $\nu(C_{ring}\text{-}C_{methyl})$ with $\rho(C_{ring}H)$ mode and the peak at 1421 cm^{-1} is assigned to $\nu(CH_3)$ mode.

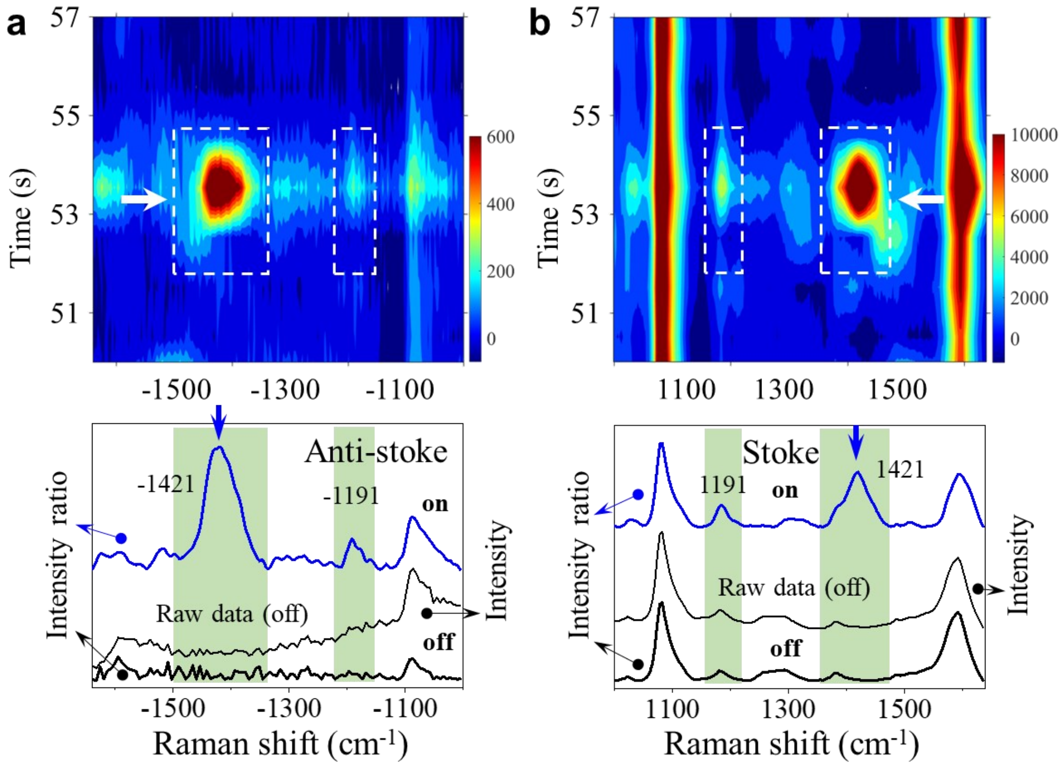

Fig. S9. (a-b) Representative SERS time trajectory containing signal-2 for anti-stoke (a) and stoke (b) spectra. The exposure time is set to be 1 s. Stacked transient spectra (after baseline correction as described in section s3, with intensity ratio y axis at the left side) at "on" and "off" states taken from the heatmaps are shown at the bottom. For comparison, the raw spectrum at the "off" state before baseline correction is also shown.

11. The local electric field enhancement calculation for 4-MBT and the 4-DMBT anion

To confirm the picocavity origin of the dynamic signals that appeared in the experiments and investigate the mechanism of the peaks appearing around 1500 cm^{-1}, we carried out the LEFE

calculations for two complex structures: Au5/4-MBT/Au2 (Fig. S10b) and Au5/4-DMBT(anion)/Au2 (Fig. S10d). As shown in the figures of the top row, the vibration mode near 1506 cm^{-1} ($\nu(C_{ring}{\approx}C_{methylene})$) only appeared for Au5/4-DMBT(anion)/Au2 structure but not for Au5/4-MBT/Au2 structure. When we place the hot spot near the (deprotonated) methyl group (Fig. S10c (ii) and (iii)), the 1506 cm^{-1} peak is greatly enhanced for the 4-DMBT anion. In contrast, multiple peaks including $\nu(C_{ring}\text{-}S)$ and $\nu(C_{ring}\text{-}C_{ring})$ are enhanced when the hot spot is placed near the benzene ring ((iv)).

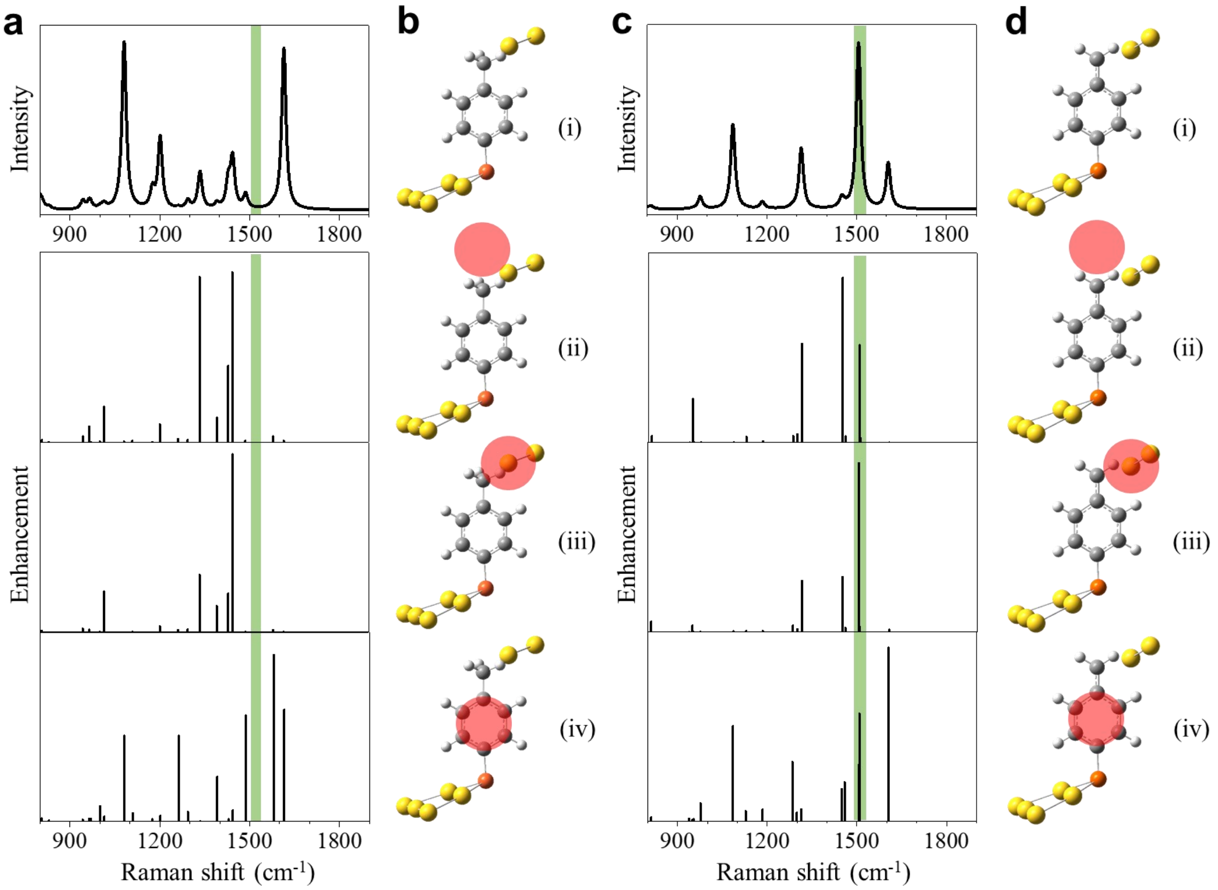

Fig. S10. (a) DFT calculation of the Raman spectra of Au5/4-MBT/Au2 complex without ((i)) and with the LEFE calculations ((ii)-(iv)). (b) Corresponding structures and the hot spot position. (c) DFT calculation of the Raman spectra of Au5/4-DMBT(anion)/Au2 complex without ((i)) and with the LEFE calculations ((ii)-(iv)). (d) Corresponding structures and the hot spot position.

12. The interaction of methyl-gold and methylene-gold

To compare the interacting strength between Au atoms and 4-MBT or 4-DMBT anion, we calculated the binding energies of two structures Au5/4-MBT/Au2 and Au5/4-DMBT/Au2. With all the atoms relaxed in both structures, we found the Au-C distances are 2.9 Å and 2.1 Å, respectively for 4-MBT and 4-DMBT. To understand the effect of Au-C distance on the binding energy, we also fixed the Au-C distance at different values and then optimized the Au5/4-DMBT/Au2 structure. Table S2 lists the calculated binding energies at different Au-C distances. The binding energy of $-CH_2^-$/Au (with Au-C distance of 3.1 Å) is more than five times higher than that of $–CH_3$/Au (with Au-C distance of 2.9 Å). The interaction strength of $–CH_3$/Au is much weaker than that of $-CH_2^-$/Au.

Table S2. Binding energies between 4-MBT or 4-DMBT anion and gold atoms

Structure	Au-C distance (Å)	Binding energy (kcal/mol)
Au5/4-MBT/Au2	2.9	-6.9
Au5/4-DMBT(anion)/Au2	2.1	-80.0
	3.1	-38.0
	4	-15.9
	4.4	-10.9
	4.9	-7.1

13. The representative SERS signals under different laser power densities

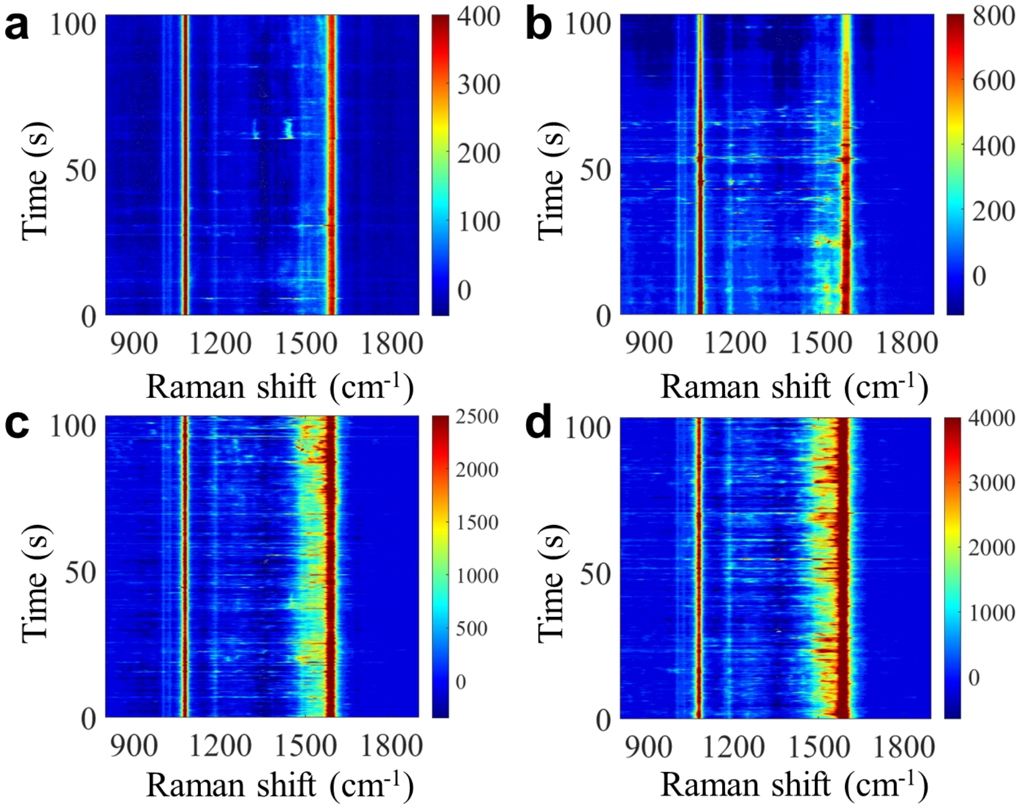

Fig. S11. (a-d) Four representative SERS trajectories in intensity heatmap format under four different laser power densities 4.6 μW/μm^2 (a), 8.14 μW/μm^2 (b), 22.65 μW/μm^2 (c), and 36.45 μW/μm^2 (d), respectively.

14. The statistical results of the fluctuating signal-2 events under different laser intensities

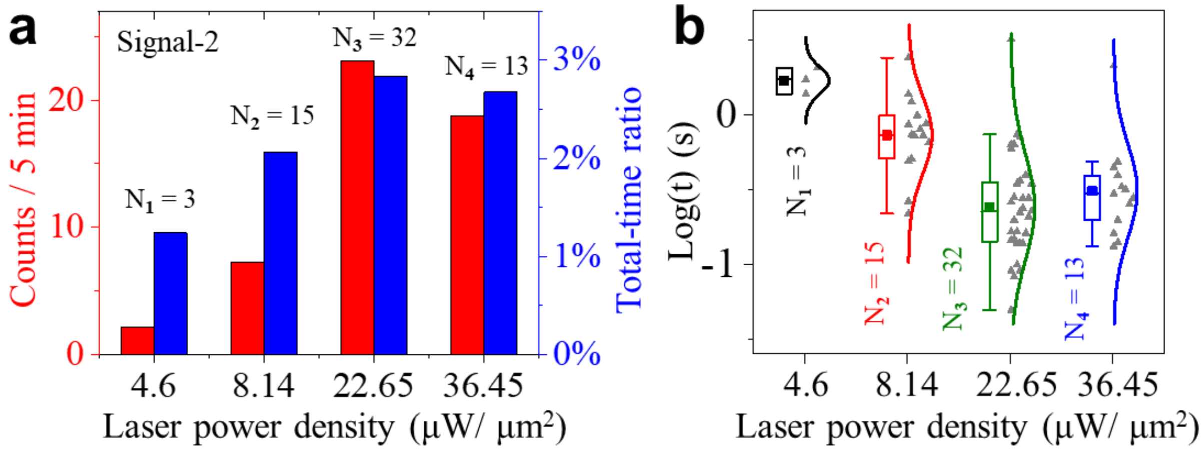

Fig. S12. (a) Statistical results of total-time percentage and counts for signal-2 under different laser intensities: 4.6, 8.1, 22.7, and 36.5 μW/μm^2. (b) Statistical results of the lifetime for signal-2 under four different laser intensities.

We also carried out the statistical analysis for the occurrence and the lifetime of the signal-2 of signals under different excitation laser intensities. Fig. S12a shows the statistical results of signal-2 for total-time percentage and frequency of occurrence under different laser intensities. We found the occurrence of signal-2 is proportional to the laser power density until saturates at 22.7 μW/μm^2. This result indicates that the formation of gold adatoms and picocavities is affected to a large extent by the intensity of incident light. Fig. S12b shows the lifetime of fluctuating events with and without the peak of the $\nu(C_{ring}{\approx}C_{methylene})$ mode of 4-DMBT anion. From the results, we found that signal-2 is more frequent and short-lived when the laser power density is higher but there is a substantial rise in the average lifetime when the laser power density is up to 22.7 μW/μm^2.

15. Control experiments in the air and ethanol solution

To understand the effect of water, we performed control experiments to compare the results both collected in the aqueous solution and in the air. As shown in Fig. S13 a, the SERS trajectory is very stable in the air, and we did not observe any dynamic fluctuations. However, after placing the same GNE in the aqueous solution, dynamic SERS signals (including signal-1 to signal-4)

appear. The most notable fluctuations are around 1530 cm^{-1} (signal-1) as shown in Fig. S13 b. We then dried the GNE with argon gas and conducted SERS measurements in the air again. The spectra became stable again, as shown in Fig. S13 c.

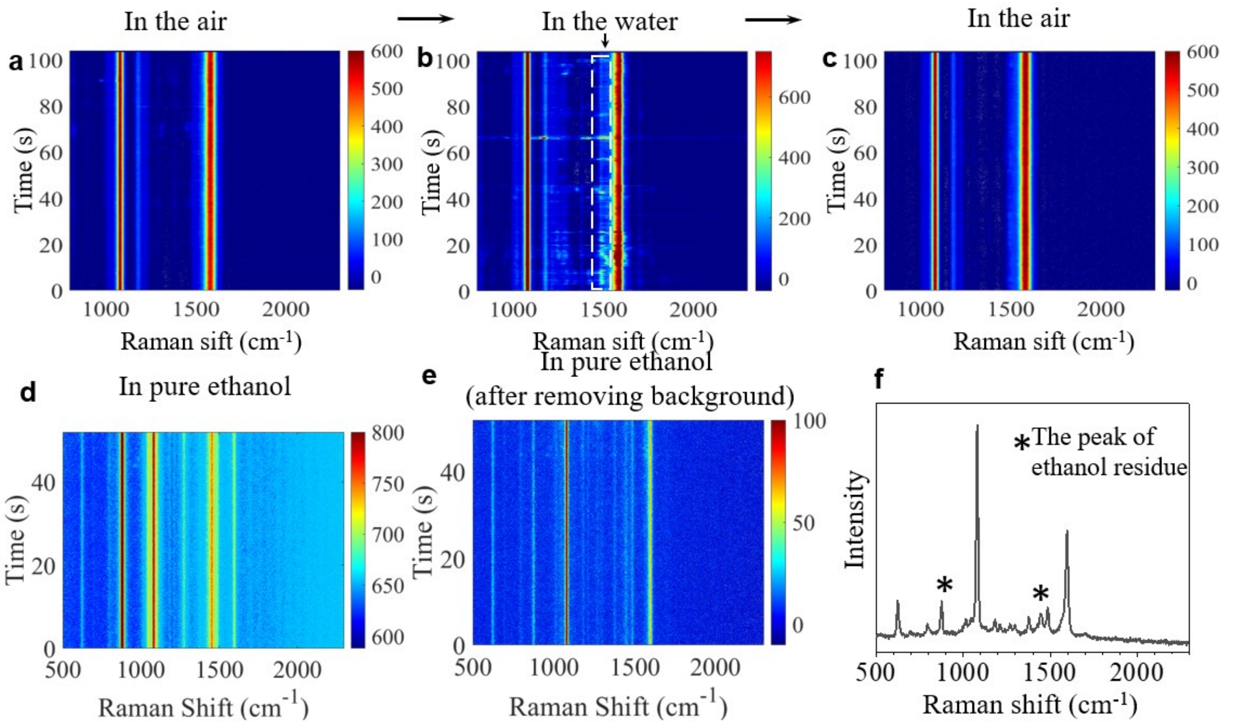

Fig. S13. (a-c) Representative SERS time trajectory of a 4-MBT modified GNE in the air (a), aqueous solution (b), and air again (c). (d-f) Experiment in absolute ethanol solution. (d) Representative SERS trajectory with the laser beam focused on the surface of GNE apex. (e) Difference SERS trajectory obtained by subtracting the average ethanol background spectrum from the SERS trajectory in (d). (f) Average SERS spectrum from the trajectory in (e). The peaks with "*" are from ethanol.

We have also conducted the control experiment at the same condition in absolute ethanol solution instead of aqueous solution. Compared with water solution, the ethanol solution is less polar, with a lower dielectric constant, and weaker hydrogen bonding. The results are shown in Fig. S13 (d-f). It is important to note that ethanol molecules also contribute Raman signals in the same spectral range below 2000 cm^{-1}. Fig. S13d shows the SERS trajectory of a 4-MBT modified GNE in absolute ethanol. Fig. S13e shows the corrected SERS trajectory after removing the ethanol background. Figure S13f shows the averaged spectrum from (e). We did not observe any burst signals in the trajectory. We also did not observe a broad peak near 1530

cm^{-1} in the average spectrum. Therefore, we conclude that the aqueous environment is important for the dehydrogenation reaction (also the following dimerization reaction) of the aromatic methyl group.

16. The inductive effect lowers the cleavage energy for the 4-MBT dehydrogenation reaction

To understand the inductive effect in the dehydrogenation reaction of 4-MBT methyl group, we compared the hydrogen atom cleavage energies in the dehydrogenation reactions (in water) between Au5/4-MBT and ethane (see Fig. S14). The cleavage energies of 4-MBT and ethane are 2.63 eV (60.65 kcal/mol) and 3.88 eV (89.47 kcal/mol), respectively. In the 4-MBT structure, the inductive effect causes the electron cloud of the methyl group shifts towards the benzene ring, thus weakening the C-H bond of the methyl group. But in the ethane molecule, the atoms are all σ-bonded and there is no conjugation and thus no inductive effect. So the C-H bond of the ethane methyl group is much stronger. This suggests that the inductive effect in 4-MBT weakens the C-H bond in the methyl group and reduces the energy required for the dehydrogenation reaction.

Dehydrogenation

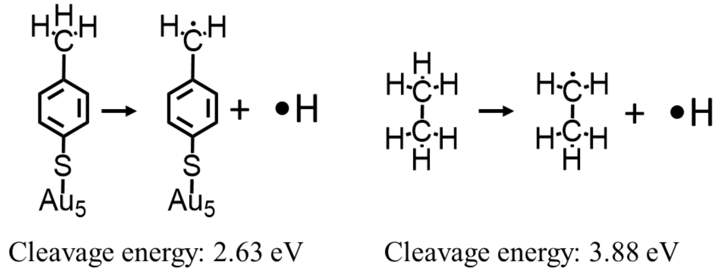

Fig. S14. The chemical reaction formula for the deprotonation and dehydrogenation of Au5/4-MBT and ethane.

17. Electrode potential (E) modulation for 4-MBT SERS signals

To understand the effect of applied E on the charge distribution and shift of 4-MBT molecules, we calculated the charge distributions in the Au5/4-MBT/Au2 and Au5/BT/Au2 structures. To account for different Es, we replaced one or two gold atoms of Au5 cluster with F or Li atoms. This is because the F atom has a strong electron-withdrawing ability while the Li atom has a strong electron-donating ability. Specifically, the Au3F2 cluster is to simulate the GNE under positive E,

the Au4Li1 cluster is to simulate the GNE under negative E, and the Au5 cluster is to simulate GNE at zero bias.

Fig. S15 shows the total charge density distribution of BT (a) and 4-MBT (b) structures. The electron-rich area is red and the electron-poor area is blue. In the structure with Au3F2(positive E), the charge of the molecule shifts towards the Au cluster. In the structure with Au4Li1(negative E), the charge density is higher on the molecule. This calculation result can well explain the experimental data in Fig. 4a.

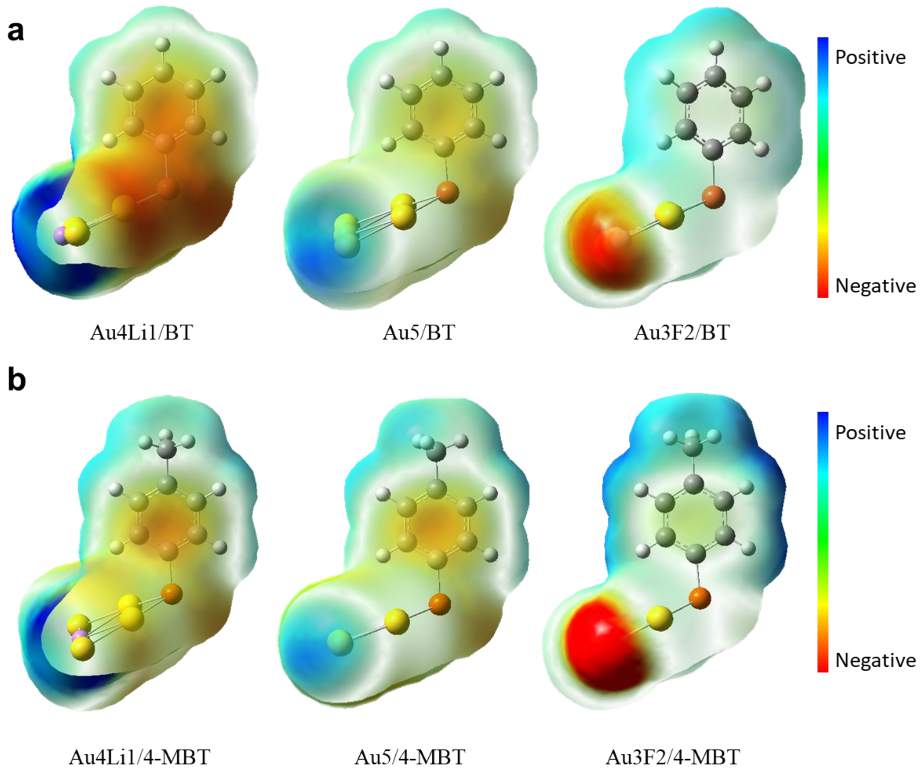

Fig. S15. Total charge density distribution of BT and 4-MBT systems by DFT calculation. (a) Total charge density distribution of Au3F2/BT, Au5/BT, and Au4Li1/BT. (b) Total charge density distribution of Au3F2/4-MBT, Au5/4-MBT, and Au4Li1/4-MBT.

Table S3. The NBO (natural bond orbital) and Mulliken charge distribution for three structures

Type	Structure	Methyl group	CP ring	Δ methyl-CP
	Au4Li1/4-MBT	0.035	-0.133	0.168
NBO	Au5/4-MBT	0.035	-0.13	0.165
	Au3F2/4-MBT	0.04	-0.112	0.152

Mulliken	Au4Li1/4-MBT	0.111	-0.12	0.231
	Au5/4-MBT	0.112	-0.11	0.222
	Au3F2/4-MBT	0.121	-0.085	0.206

Table S3 lists the NBO and Mulliken charge of three structures which are Au4Li1/4-MBT, Au5/4-MBT, and Au3F2/4-MBT, respectively. We have listed the charges of the methyl and benzene rings and the charge difference between them, the smaller this charge difference is, the less the polarization is. Based on the results, we find that the presence of the F atom lowers the charge of the benzene ring, thus lowering the charge difference between the benzene ring and the methyl group, which makes it easier to transfer the charge of the methyl group to the benzene ring under an inductive effect, thus making the dehydrogenation reaction easier.

18. The evidence of nanogap distance modulation by electrode potential (E)

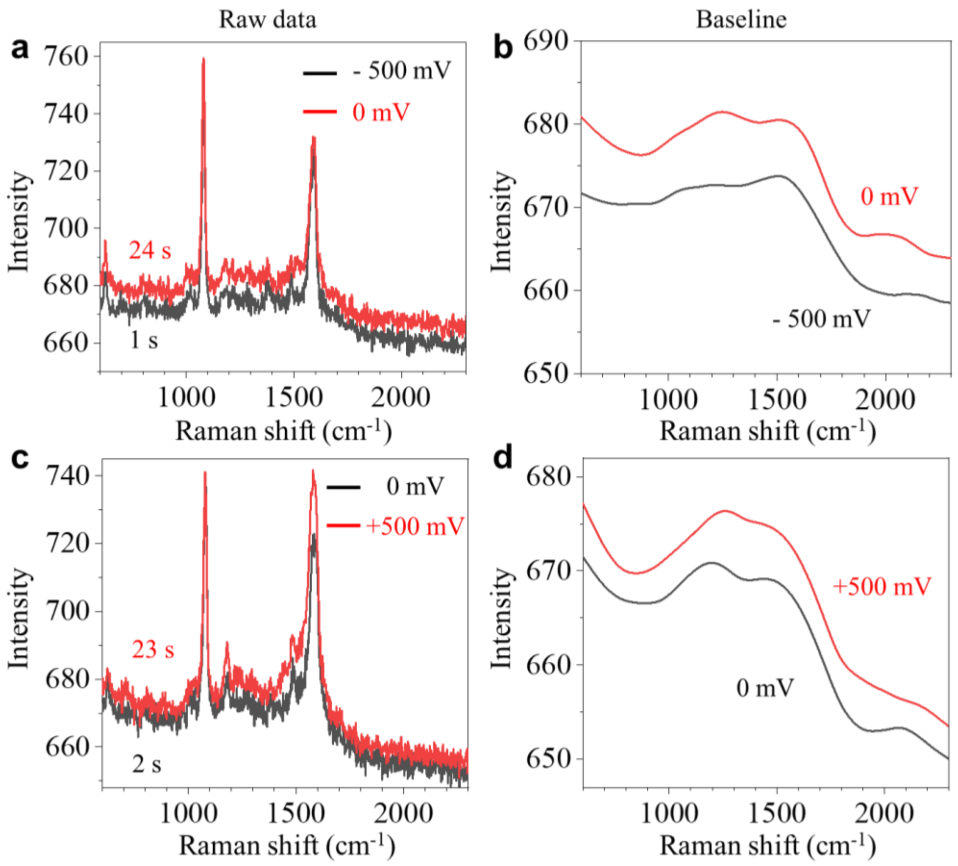

Fig. S16. (a) Two spectra before baseline correction with applied E from -500 mV (black spectrum) to 0 mV (red spectrum). (b) The baseline of the two spectra in (a). (c) Two spectra before baseline correction with applied E from 0 mV to +500 mV. The black line is for 0 mV and the red line is for +500 mV. (d) Baselines of two spectra from (c). For both cases, the E was switched right after taking the first spectrum (black color one).

When applied E to the GNEs, we usually noticed obvious changes in the baseline intensity of the SERS spectra. We further conducted control experiments to confirm this phenomenon. The experiments were repeated on three GNEs and different locations on each GNE. At each location, we switch E from -500 mV to 0 mV and then from 0 mV to +500 mV. The observed baseline intensity change pattern was essentially the same. One representative result is shown in Fig. S16. When the E was switched from -500 mV to 0 mV (Fig. S16 a and b) or from 0 mV to +500 mV (Fig. S16 c and d), the baseline intensity gradually but not immediately increased in about 20 seconds. Importantly, the decrease of intensity at the negative E is opposite to the typically observed intensity increase trend of the molecule vibration peaks, which may be caused by Fermi level shift and hydrogen evolution.[8]

The SERS background originates from the localized surface plasmon resonance (LSPR)-modulated photoluminescence[9] and is sensitive to the change of plasmonic structure. One possible mechanism for the gradual change is the E-induced electromechanical changes of the NPoNE structures.[3] With the change of E towards a positive direction, the negatively charged GNPs are forced to move closer to the GNE in response to the electrostatic force thus reducing the gold nanogap and enhancing the baseline intensity.

19. The effects of applied E and nanogap distance for 4-DMBT anion signals

To investigate the effects of applied E and the nanogap distance change to the 4-DMBT anion signals, we calculated five structures, which are Au3F2/4-DMBT/Au2, Au4F1/4-DMBT/Au2, Au4Li1/4-DMBT/Au2, and Au4Li1/4-DMBT, Au5/4-DMBT/Au2. The structures of the first four are shown in Fig. S17. We defined the Au-C distance as the interaction distance between the methylene group of 4-DMBT anion and Au2 cluster, which are 4.05 Å (a), 4.39 Å (b), and 5.46 Å (c), respectively. Fig. S17 d shows the Au4Li1/4-DMBT structure without an Au2 cluster, which

represents the case without interaction. The F and Li atoms are used to mimic the electron shift induced by the applied E. The local electric field enhancement calculation (LEFE) is carried out based on the interacting gold atom of the Au2 cluster to simulate the picocavity effect.

Fig. S17 also shows the calculated normal Raman spectra and the modified vibration modes after LEFEs. The signature peak of $\nu(C_{ring}{\approx}C_{methylene})$ for 4-DMBT anion is greatly enhanced by the nearby picocavity. The wavenumber of this peak became bigger (blue shift) with the increase of Au-C distance from Fig. S17 a to d, revealing the impacts of the Au- methylene interaction. It is worth noting that other peaks may be enhanced as well in the range between 1200 cm^{-1} and 1500 cm^{-1}, which were also observed experimentally.

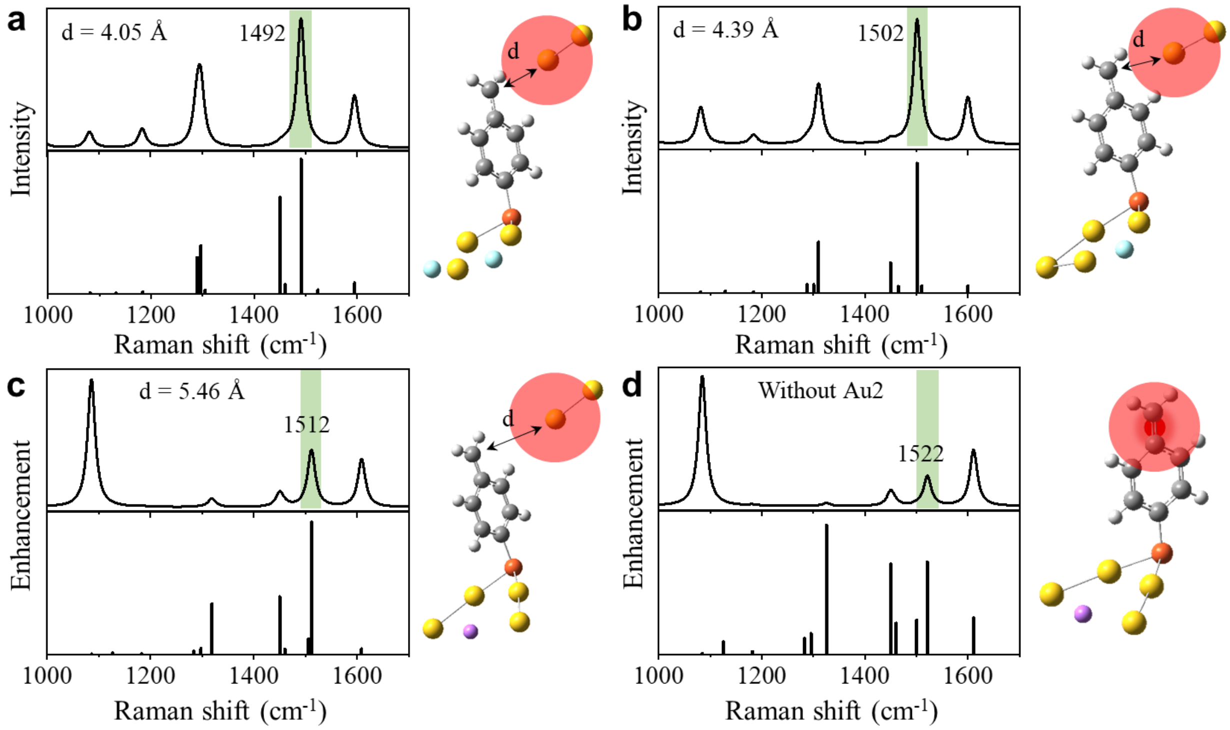

Fig. S17. DFT calculations of the Raman spectra and the enhanced vibration modes (vertical lines below the spectra) by picocavity through LEFEs for four structures: (a) Au3F2/4-DMBT/Au2 structure; (b) Au4F1/4-DMBT/Au2 structure; (c) Au4Li1/4-DMBT/Au2 structure; (d) Au4Li1/4-DMBT structure. The picocavity positions are indicated as red circles on the structures. The spectral range is shown from 1000 cm^{-1} to 1700 cm^{-1}.

20. The calculated energies in the dimerization reaction

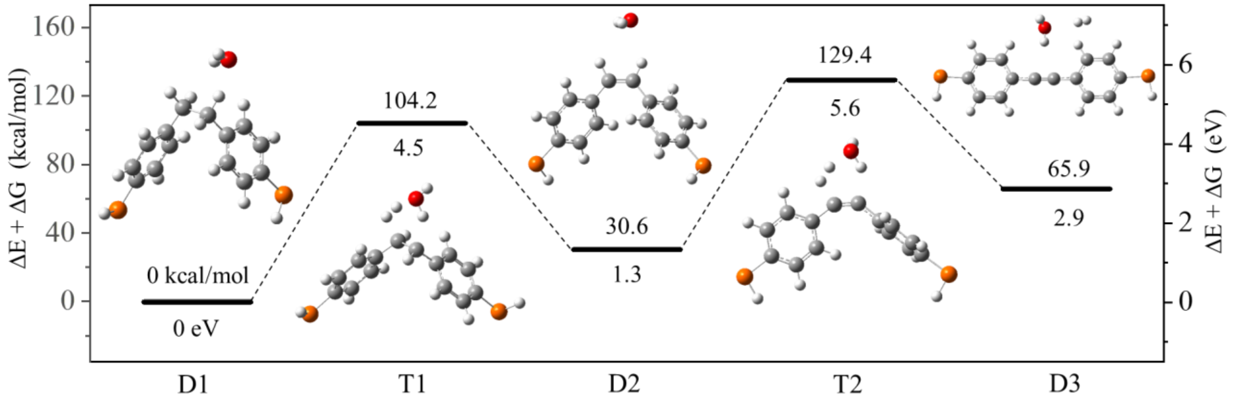

Fig. S18. DFT calculated relative energies (ΔE+ΔG) for the reaction pathway from D1 to D3.

The dimerization reaction pathway is shown in Fig. 5c of main text. As we discussed in section S7 (Fig. S6), the energy required to cleave a proton radical (hydrogen atom) from 4-MBT methyl group is 60.8 kcal/mol (2.63 eV). Fig. S18 shows the DFT calculated energies for the intermediates and transition states. The relative energies (ΔE+ΔG) are the sums of electronic (ΔE) and thermal free energies (ΔG) calculated at 298.15 K.

21. The wagging vibration mode of methylene group in 4-DMBT

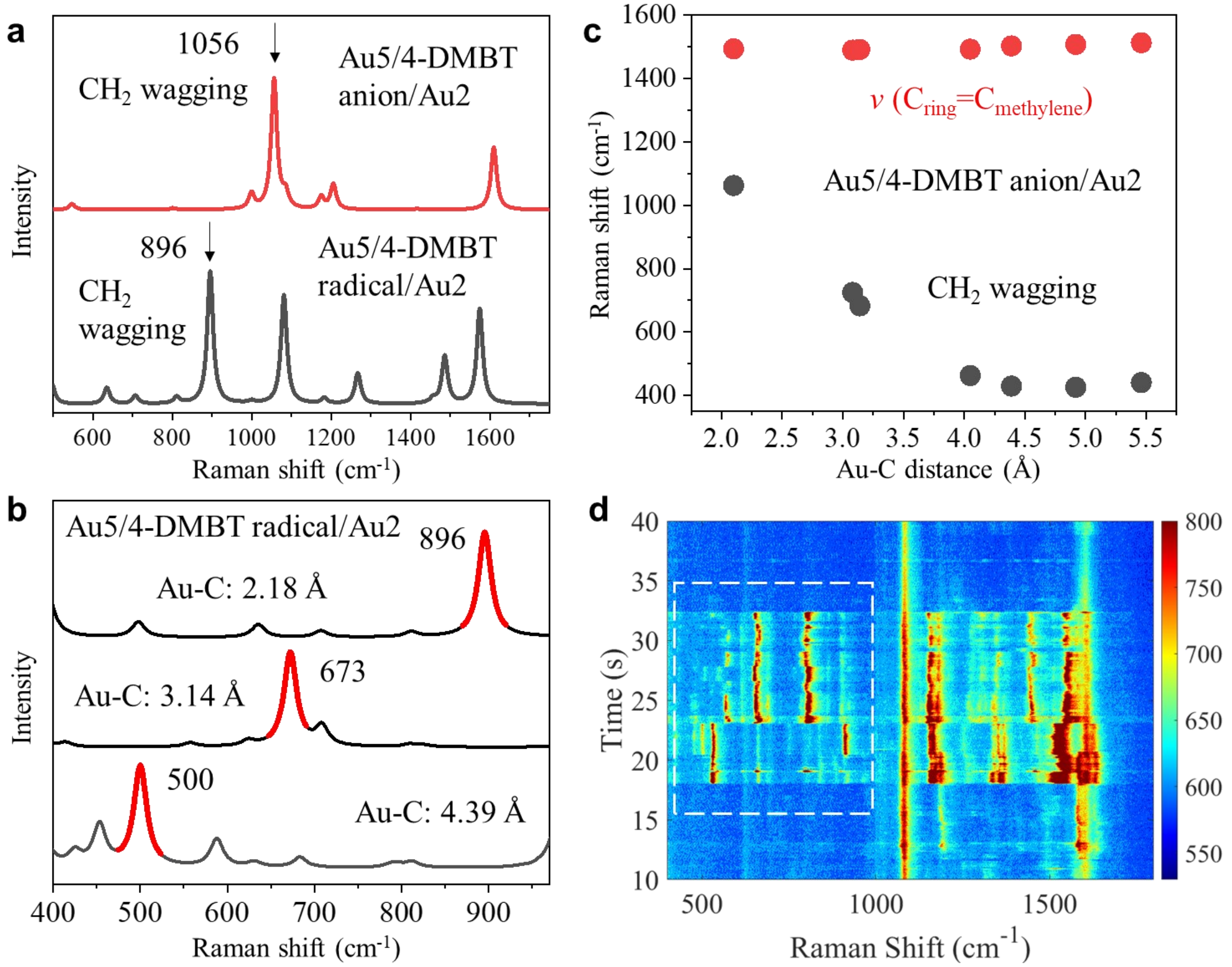

Fig. S19. (a) DFT calculated spectrum of Au5/4-DMBT radical/Au2 (black) and Au5/4-DMBT radical/Au2 (red) structures. (b) Three stacked spectra of Au5/4-DMBT radical/Au2 structure with different Au-C distances. The peak of CH_2 wagging mode is highlighted in red. (c) Peak positions of CH_2 wagging and $v(C_{ring}{\approx}C_{methylene})$ modes as a function of Au-C distance in the Au5/4-DMBT anion/Au2 structure. (d) Representative transient SERS trajectory with peaks both near 1530 cm^{-1} and in the range of 400 cm^{-1} ~ 900 cm^{-1}.

Based on the complex Au5/4-DMBT radical (or anion)/Au2, we studied the wagging vibration mode of the methylene group (see Fig. S19a) that has been reported before.[10, 11] This mode is strongly affected by the interaction between Au and Methyelene group and very sensitive to the Au-C (of the methylene group) distance. Taking the Au5/4-DMBT radical/Au2 structure as an

example (Fig. S19b), with the Au-C distance near 2.18 Å, this vibration mode is near 896 cm^{-1}. When the Au-C distance is increased to 3.14 Å, the peak position moves to 673 cm^{-1}. At Au-C distance 4.39 Å, the peak position is near 500 cm^{-1}. In Fig. S19c, we compared the peak position of CH_2 wagging and $\nu(C_{ring} \approx C_{methylene})$ modes as a function of Au-C distance in the structure of Au5/4-DMBT anion/Au2. The wavenumber of CH_2 wagging peak varied significantly from 400 cm^{-1} to nearly 1100 cm^{-1} over a range of Au-C distances from 2.0 Å to 5.5 Å. In contrast, the vibration mode of methylene group ($\nu(C_{ring} \approx C_{methylene})$) near 1530 cm^{-1} is far less sensitive to the Au-C bond distance. Over the same Au-C distance range, the wavenumber of $\nu(C_{ring} \approx C_{methylene})$ peak is confined in the small range 1500 ~ 1530 cm^{-1}. Meanwhile, 4-MBT does not have vibration modes between 1500-1530 cm^{-1}. Therefore, the peak of $\nu(C_{ring} \approx C_{methylene})$ mode can better serve as the signature peak. In Fig. S19d, we show a representative SERS trajectory with peaks both near 1530 cm^{-1} and in the range of 400 cm^{-1} ~ 900 cm^{-1}. The sudden increase of SERS intensity between time 17-33 s is attributed to a picocavity. It is difficult to determine if a peak is from the CH_2 wagging mode considering the Au-C distance may vary a lot in a stochastic picocavity.

22. The influence of oxygen on 4-MBT dehydrogenation

Considering the 4-MBT dehydrogenation reaction is an oxidation reaction, we also investigated the influence of dissolved oxygen molecule on 4-MBT dehydrogenation reaction by conducting a controlled experiment. We used argon or air bubbling to modulate the level of dissolved oxygen in the solution of liquid cell. First, we conducted air bubbling to ensure the presence of dissolved oxygen in the solution. We then conducted SERS measurements for 10 min. Next, we conducted argon bubbling to remove the dissolved oxygen in the solution. After every five SERS measurements (total about 10 min), we conducted argon bubbling again to keep the level of dissolved oxygen low.

We repeated the experiments three time and the same trend was observed. We carried out the statistical analysis for the occurrence and the lifetime of signal-1 from two experiments with the most signals. Fig. S20 shows the occurrence frequency (per 10 min) and the time ratio (total occurrence time of signal-1/total measurement time) during air bubbling and argon bubbling in the two experiments. After the argon bubbling, there are about 20-30% drop in the occurrence

frequency (per 10 min) of signal-1. The time ratio follows the same trend. Although the occurrence of signal-1 is reduced after oxygen removal, there are still enough signals being observed. In addition, we still observed the peak near 2150 cm^{-1} after argon bubbling. Therefore, we concluded that the plasmon activated 4-MBT dehydrogenation in aqueous solution can proceed without oxygen but the presence of oxygen can indeed promote 4-MBT dehydrogenation.

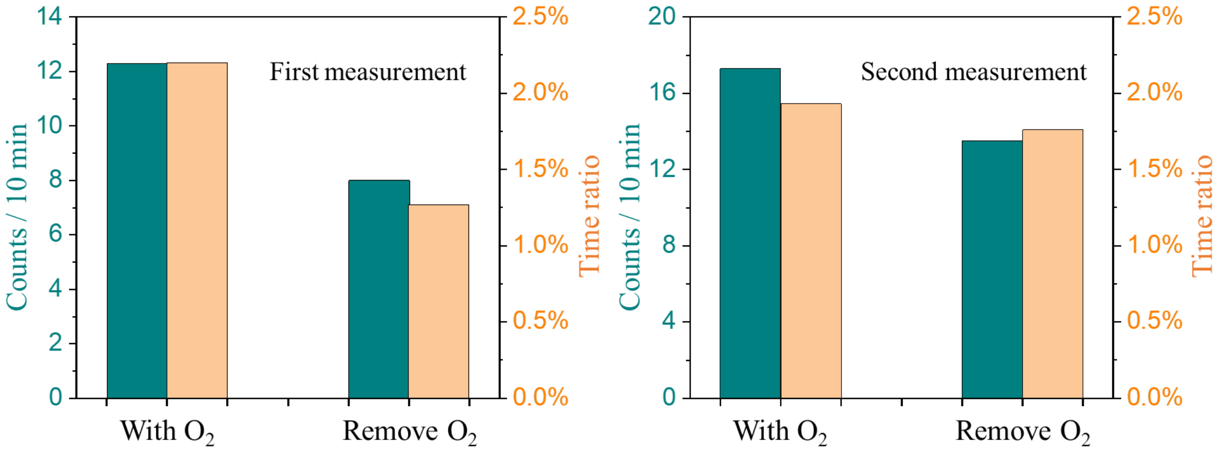

Fig. S20. The occurrences per 10 minutes (dark cyan) and the time ratio (orange) of signal-1 (the peak near 1530 cm^{-1}) for two repeated experiments. The total measurement time for the first experiment is 32 min (after air bubbling) and 36 min (after argon bubbling). The total measurement time for the second experiment is 71 min (after air bubbling) and 55 min (after argon bubbling).

23. DFT calculation of 4-MBT dehydrogenation with the involvement of oxygen

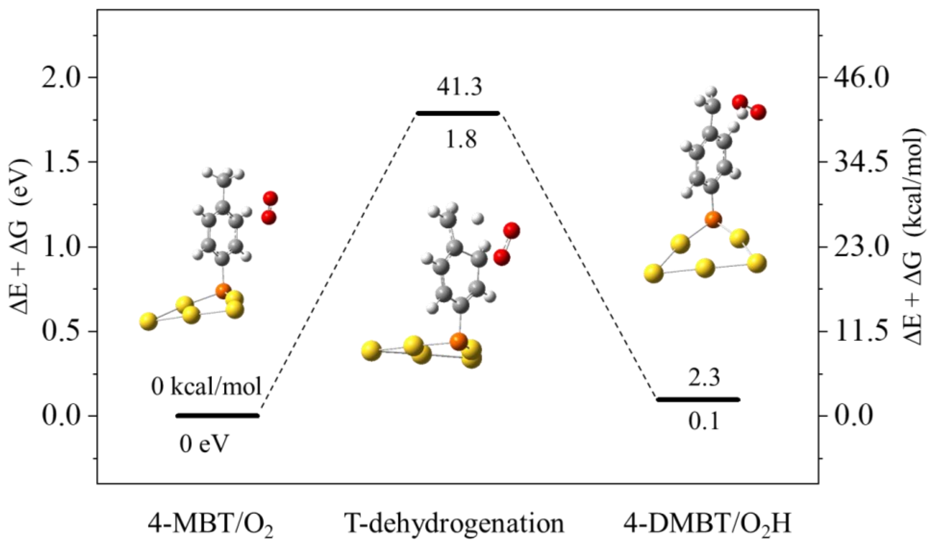

Fig. S21. DFT calculated relative energies (ΔE+ΔG) for the dehydrogenation reaction with oxygen involved. The 4-MBT/O_2 is the structure of 4-MBT adsorbing oxygen. The T-dehydrogenation is the transition state of this oxidative dehydrogenation reaction. The 4-DMBT(radical)/•O_2H is the structure of 4-DMBT (radical) with •O_2H radical after dehydrogenation.

We conducted DFT calculations to explore the possible reaction pathway for the oxygen molecule participated 4-MBT dehydrogenation reaction. One proposed reaction pathway is shown in Figure S21. We have successfully calculated the transition state of this reaction. Initially, oxygen molecule interacts with the hydrogen atom of the methyl group of 4-MBT. Subsequently, oxygen molecule acquires the hydrogen atom leaving the methyl group at the transition state (T-dehydrogenation), resulting in the formation of an •O_2H radical and 4-DMBT radical. Figure S21 also shows the calculated energies. The interaction with the oxygen molecule can greatly reduce the energy barrier from 2.63 eV (Figure S6) to 1.8 eV to cleavage hydrogen atom.

References:

1. J. Guo, J. Pan, S. Chang, X. Wang, N. Kong, W. Yang and J. He, Monitoring the Dynamic Process of Formation of Plasmonic Molecular Junctions during Single Nanoparticle Collisions, *Small*, 2018, **14**, e1704164.
2. J. Zhou, J. Guo, A. M. Mebel, G. Ghimire, F. Liang, S. Chang and J. He, Probing the Intermediates of Catalyzed Dehydration Reactions of Primary Amide to Nitrile in Plasmonic Junctions, *ACS Catalysis*, 2022, DOI: 10.1021/acscatal.2c01793, 7737-7747.
3. T. Ma, J. Guo, S. Chang, X. Wang, J. Zhou, F. Liang and J. He, Modulating and probing the dynamic intermolecular interactions in plasmonic molecule-pair junctions, *Phys Chem Chem Phys*, 2019, **21**, 15940-15948.
4. Q. Ai, J. Zhou, J. Guo, P. Pandey, S. Liu, Q. Fu, Y. Liu, C. Deng, S. Chang, F. Liang and J. He, Observing dynamic molecular changes at single-molecule level in a cucurbituril based plasmonic molecular junction, *Nanoscale*, 2020, **12**, 17103-17112.
5. N. Kong, J. Guo, S. Chang, J. Pan, J. Wang, J. Zhou, J. Liu, H. Zhou, F. M. Pfeffer, J. Liu, C. J. Barrow, J. He and W. Yang, Direct Observation of Amide Bond Formation in a Plasmonic Nanocavity Triggered by Single Nanoparticle Collisions, *J Am Chem Soc*, 2021, **143**, 9781-9790.
6. M. Inagaki, T. Isogai, K. Motobayashi, K. Q. Lin, B. Ren and K. Ikeda, Electronic and vibrational surface-enhanced Raman scattering: from atomically defined Au(111) and (100) to roughened Au, *Chem Sci*, 2020, **11**, 9807-9817.
7. F. Benz, M. K. Schmidt, A. Dreismann, R. Chikkaraddy, Y. Zhang, A. Demetriadou, C. Carnegie, H. Ohadi, N. B. De and R. Esteban, Single-molecule optomechanics in "picocavities", *Science*, 2016, **354**, 726-729.
8. J. Guo, G. Ghimire, J. Zhou, L. Yu, Z. Wang, S. Chang and J. He, Potential Controlled Redox Cycling of 4-aminothiophenol by Coupling Plasmon Mediated Chemical Reaction with Electrochemical Reaction, *J. Catal.*, 2023, DOI: https://doi.org/10.1016/j.jcat.2023.01.018.
9. K.-Q. Lin, J. Yi, J.-H. Zhong, S. Hu, B.-J. Liu, J.-Y. Liu, C. Zong, Z.-C. Lei, X. Wang, J. Aizpurua, R. Esteban and B. Ren, Plasmonic photoluminescence for recovering native chemical information from surface-enhanced Raman scattering, *Nature Communications*, 2017, **8**, 14891.
10. Y.-F. Huang, D.-Y. Wu, A. Wang, B. Ren, S. Rondinini, Z.-Q. Tian and C. Amatore, Bridging the Gap between Electrochemical and Organometallic Activation: Benzyl Chloride Reduction at Silver Cathodes, *Journal of the American Chemical Society*, 2010, **132**, 17199-17210.
11. A. Wang, Y.-F. Huang, U. K. Sur, D.-Y. Wu, B. Ren, S. Rondinini, C. Amatore and Z.-Q. Tian, In Situ Identification of Intermediates of Benzyl Chloride Reduction at a Silver Electrode by SERS Coupled with DFT Calculations, *Journal of the American Chemical Society*, 2010, **132**, 9534-9536.
